# Physiology-informed LSTM framework integrating crop model and Sentinel-2 time series for rice nitrogen status estimation

**DOI:** 10.1016/j.plaphe.2026.100234

**Published:** 2026-06-05

**Authors:** Jinpeng Yang, Zhaopeng Fu, Ke Zhang, Bohuai Shi, Jiang Wang, Weizhe Zhao, Qiang Cao, Yongchao Tian, Yan Zhu, Weixing Cao, Xiaojun Liu

**Affiliations:** a National Engineering and Technology Center for Information Agriculture, Key Laboratory for Crop System Analysis and Decision Making (Ministry of Agriculture and Rural Affairs), Engineering Research Center of Smart Agriculture (Ministry of Education), Jiangsu Key Laboratory for Information Agriculture, Jiangsu Collaborative Innovation Center for Modern Crop Production, Nanjing Agricultural University, Nanjing, 210095, China; b Institute of Sanya, Nanjing Agricultural University, Sanya, 572000, China

**Keywords:** Physiology-informed LSTM, Precision nitrogen management, Plant dry matter, Plant N accumulation, Variable-rate fertilization

## Abstract

Accurate assessment of plant dry matter (PDM) and plant N accumulation (PNA) provides essential indicators for precision nitrogen (N) management in rice production. However, purely data-driven models struggle to generalize due to the spatial scarcity of ground-truth physiological data. To address this, a physiology-informed long short-term memory (PI-LSTM) framework was developed for robust regional N diagnosis and variable-rate fertilization. First, the model was pretrained to internalize crop growth dynamics using a DSSAT-based simulation library, which spanned 2000 representative fields and 700 management scenarios to provide physiologically consistent pseudo-labels. Subsequently, the framework was fine-tuned using multi-year field observations (2020, 2023, 2024), Sentinel-2 time-series data, and meteorological inputs. The proposed LSTM framework outperformed conventional machine learning approaches in estimating PDM and PNA, achieving five-fold cross-validation R^2^ values of 0.87 and 0.83, respectively. Based on these biophysical estimations, the N nutrition index (NNI) diagnosis achieved a 67.3% overall classification accuracy. Furthermore, by integrating the critical N dilution curve, the critical PNA and accumulated N deficiency (AND) were quantified, which served as the basis for developing the AND-based N recommendation algorithm (ANDA). Finally, variable-rate topdressing field experiments conducted across seven sites in 2024 and 2025 demonstrated that the ANDA reduced N input by 13.4% compared with farmers’ practices, while maintaining or increasing yield and improving N partial factor productivity by 18.6%. This study provides a reliable, physically consistent decision-support framework for regional-scale precision N management.

## 1 Introduction

Nitrogen (N) management plays a pivotal role in securing food production and promoting sustainable rice (*Oryza sativa L.*) production [1,2], yet suboptimal and uniform N application practices often lead to greenhouse gas emissions and groundwater contamination [1,3]. To address these trade-offs, variable-rate N fertilization management has emerged as a key pathway for sustainable agriculture [4]. Central to this approach is the N nutrition index (NNI), widely regarded as the gold standard for diagnosing crop N status [5]. However, its application of NNI at regional scales remains challenging because it requires continuous and accurate quantification of two fundamental biophysical variables: plant dry matter (PDM) and plant N accumulation (PNA).

Accurate large-scale inversion of PDM and PNA is crucial for in-season, real-time crop management. Currently, the mainstream methodologies for regional estimation primarily rely on two independent approaches: remote sensing-based data-driven methods and process-based crop models. Remote sensing techniques, utilizing satellite imagery and deep learning (DL), excel at capturing spatial variability and have been widely applied for regional monitoring. However, their operational application is chronically hindered by the “small sample” dilemma. Purely data-driven models require massive amounts of ground truth data, which relies on labor-intensive and spatially sparse destructive field sampling [6]. Consequently, models trained on limited datasets often suffer from overfitting and exhibit poor generalization across different growing seasons or regions [7]. Conversely, process-based models (PBMs, e.g., DSSAT, APSIM) offer a robust alternative by encapsulating physiological mechanisms to simulate crop growth under varying environments. Yet, their reliance on complex parameterization and prohibitively high computational costs makes them difficult to apply directly for real-time, pixel-level spatial prediction over large areas [8]. Consequently, neither approach alone can fully satisfy the need for physiologically credible, spatially scalable, and in-season N diagnosis under sparse field observations.

Recent advances in physiology-informed machine learning provide a promising avenue to bridge these gaps [6,9]. Specifically, transfer learning offers an effective strategy to bridge the gap between mechanistic simulations and real-world observations. By utilizing process-based crop models as 'data generators' to produce massive synthetic samples, deep learning networks can be pre-trained to internalize intrinsic biological growth trajectories, effectively learning how crops develop under varying meteorological drivers [10]. Subsequently, these pre-trained models can be fine-tuned with limited field observations to adapt to site-specific conditions. This approach not only preserves the mechanistic consistency of PBMs but also enhances the prediction accuracy in heterogeneous fields. Although process-based model-guided pre-training has recently been applied to tasks such as crop phenology and soil nitrogen simulation, most studies have relied primarily on continuous weather- and management-driven time series at point or experimental scales. However, a critical gap remains: limited attention has been paid to integrating physiological knowledge from crop models, irregular satellite time series, and transfer learning to support spatially scalable and in-season rice nitrogen diagnosis under sparse field observations.

To address these challenges, this study develops a physiology-informed LSTM (PI-LSTM) framework via a pseudo-labelling strategy for rice N diagnosis and variable-rate fertilization. The study aims to examine whether a DSSAT-informed LSTM framework can provide reliable regional estimates of PDM and PNA under sparse field observations and irregular satellite time series, and whether these estimates can support in-season rice N diagnosis and fertilization management. Specifically, the objectives are to: 1) construct a spatiotemporal estimation model for rice PDM and PNA by integrating the DSSAT mechanism with the LSTM network; 2) develop a dynamic variable-rate fertilization method based on the estimated accumulated N deficit algorithm (ANDA); 3) validate the framework at a regional scale in Xinghua City and Jiangsu Province.

## 2 Materials and methods

### 2.1 Study area and experimental design

The study was conducted in Xinghua City, Jiangsu Province, China (32.94°N, 119.83°E), a typical rice-wheat rotation region located in the lower reaches of the Yangtze River (Fig. S1). The region experiences a subtropical monsoon climate, characterized by simultaneous rain and heat during the rice-growing season, with an average annual temperature of approximately 17.3°C and annual precipitation of roughly 900 mm. Rice is typically grown from June to November. The soil in the study area is classified as Haplic Anthrosol (Endogleyic) according to the World Reference Base for Soil Resources [11]. To develop, fine-tune, and validate the proposed physiology-informed LSTM framework and its agronomic application, a comprehensive series of field experiments were conducted from 2017 to 2025 in Xinghua City. The study comprises three distinct categories of trials: controlled plot-scale experiments (Exp. 1), multi-site farm-scale surveys (Exp. 2), and N regulation validation trials (Exp. 3). First, to capture fundamental crop growth responses under varying N gradients, controlled plot-scale trials (Exp. 1) were conducted in 2017 and 2018 at Diaoyu Farm (DY, 119.91°E, 33.06°N). These trials involved two rice cultivars (Nanjing 9108 and Yongyou 2640) subjected to four N application rates (0, 135, 270, and 405 kg ha^−1^) and diverse planting methods, including direct seeding, mechanical pot transplanting, and mechanical mat transplanting.

To generate a dataset for domain adaptation and assess model robustness under real-world production conditions, multi-site farm-scale trials (Exp. 2) were conducted in 2020, 2023, and 2024. These trials spanned five distinct locations with varying soil and management conditions, including Daiyao Farm (DAY, 120.18°E, 32.95°N), Daduo Farm (DD, 120.03°E, 32.89°N), Lincheng Farm (LC, 119.80°E, 32.88°N), Zhouzhuang Farm (ZZ, 119.90°E, 32.70°N), and Diaoyu Farm. In contrast to the controlled gradients in Exp. 1, these trials followed local farmers' fertilization and planting practices to reflect the spatial heterogeneity of actual agricultural production.

Finally, to verify the practical value of the proposed variable-rate fertilization strategy, N regulation validation trials (Exp. 3) were implemented in 2024 and 2025. The 2024 trials were conducted at DY, LC, and ZZ Farms, while the 2025 validation was conducted at DY Farm, Xinwei Farm (XW, 118.29°E, 33.14°N) and Luoshe Farm (LS, 120.18°E, 31.64°N). Sampling was focused on the maturity stage to quantify final yield.

**Fig. 1:**
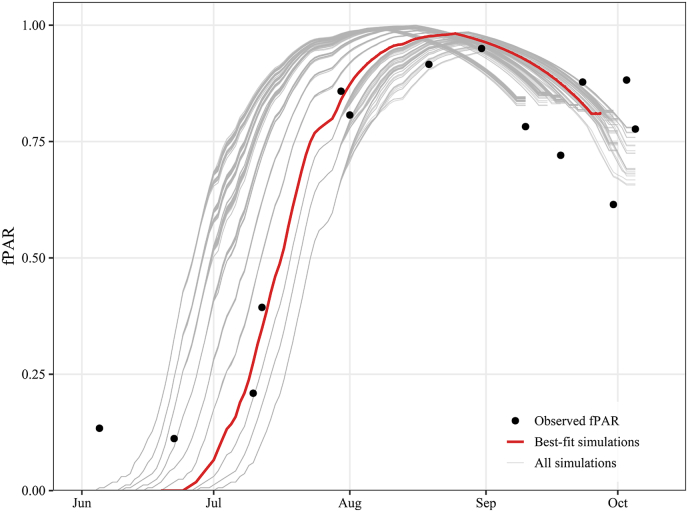
Overview of the research methodology. PDM and PNA refer to plant dry matter and plant nitrogen accumulation, respectively. NR denotes the nitrogen requirement, and AND represents the accumulated nitrogen deficiency.

Rice phenological stages were recorded using the Biologische Bundesanstalt, Bundessortenamt und Chemische Industrie scale (BBCH) [12], with the dataset purposes specific timings for destructive sampling detailed in Table 1. The overall workflow of the proposed PI-LSTM framework is summarized in Fig. 1, including field data acquisition, DSSAT-based pseudo-label reconstruction, LSTM pretraining and transfer learning, and downstream N diagnosis and recommendation.

**Table 1:** The basic information of each rice experiment.

Experiment No.	Experiment type	Year	Experiment site	Cultivar	Planting method	N rate (kg·ha^−1^)	Planting date	Sample date
Exp 1	Plot-scale trial (DSSAT calibration)	2017	DY Farm	Nanjing 9108, Yongyou 2640	MPT, MMT, DS	0, 135, 270, 405	Jun 14	BBCH: 30, 43, 55, 70, 92
		2018		Nanjing 9108, Yongyou 2640	MPT, MMT	0, 135, 270, 405	Jun 18	BBCH: 25, 30, 43, 55, 70, 92
Exp 2	Multi-site farm-scale dataset (Model fine-tuning)	2020	DAY Farm, DD Farm, DY Farm	Nanjing 9108	MPT	Local fertilizer application	Local planting date	BBCH: 26, 30, 32, 43
		2023	DY Farm					
		2024	DY Farm, LC Farm, ZZ Farm					
Exp 3	Nitrogen regulation validation trial	2024	DY Farm, LC Farm, ZZ Farm	Nanjing 9108	MPT	Local fertilizer application	Local planting date	BBCH: 92
		2025	DY Farm, XW Farm, LS Farm					

Note: MPT refers to mechanical pot transplanting, MMT to mechanical mat transplanting, DS to direct sowing, and MT to manual transplanting. The BBCH scale denotes growth stages as defined by the Biologische Bundesanstalt, Bundessortenamt, and Chemical Industry scale. “Local planting date” refers to the conventional transplanting window recommended for the local area, approximately from early to late June (June 1-30).

### 2.2 Data acquisition and preprocessing

#### 2.2.1 Ground data acquisition

Destructive sampling was performed to determine above-ground biomass and N content. At each sampling point, representative rice plants were collected and separated into stems and leaves. Samples were oven-dried at 105°C for 30 min to de-enzyme, followed by drying at 80°C to a constant weight to measure PDM. Dried samples were then milled, and the plant N concentration (PNC) was determined using the standard Kjeldahl method. Finally, PNA was calculated as the product of PDM and PNC. At the maturity stage, rice grain yield was determined using a quadrat sampling method (1 × 1 m^2^). Three representative sampling points were selected within each experimental plot or field. The harvested samples were naturally air-dried, threshed, and weighed to determine the actual grain yield.

#### 2.2.2 Meteorological data

Daily meteorological data covering the years 2017-2018 and 2020-2024 were obtained from the China Meteorological Data Sharing Service System (https://data.cma.cn/). The 2017-2018 field experiments in Exp. 1 were used for model calibration and validation, whereas the 2020-2024 data were used to drive regional DSSAT simulations for pseudo-label generation (Section 2.3). The dataset includes key variables driving crop growth: daily maximum temperature (MAX), daily minimum temperature (MIN), precipitation (RAIN), and solar radiation (SAR). These data were initially acquired as station-based observations. To facilitate regional-scale analysis, the station data were spatially interpolated using the ordinary kriging method to generate a continuous gridded dataset with a spatial resolution of 10 km × 10 km. Subsequently, this gridded meteorological dataset served as input to drive DSSAT simulations for each grid cell and generate the corresponding regional pseudo-label scenario library (detailed in Section 2.3).

#### 2.2.3 Satellite imagery and vegetation indices

Remote sensing data were acquired from the Sentinel-2 Multi-Spectral Instrument archive via Google Earth Engine. Level-2A bottom-of-atmosphere surface reflectance products (2020-2024 rice-growing seasons) were used. To ensure data quality, a strict cloud masking procedure was applied using the QA60 band to filter out pixels contaminated by clouds and shadows.

Field boundaries for the rice paddies in Xinghua City were derived from a previous study [13]. For each Sentinel-2 acquisition, vegetation indices (VIs) were calculated using combinations of relevant spectral bands, including the normalized difference vegetation index (NDVI) [14], normalized difference red edge index (NDRE) [15], red edge soil-adjusted vegetation index (RESAVI) [16], chlorophyll index red edge (CIRE) [17], and green normalized difference vegetation index (GNDVI) [18]. These indices were then aggregated to the field scale by taking the median value of all valid 10 m pixels within each field polygon. The 10 km × 10 km meteorological grids were used to represent regional weather forcing for DSSAT simulations, while Sentinel-2 variables were aggregated to the field scale to match the spatial support of field-level pseudo-label reconstruction. To balance computational efficiency with regional representativeness, approximately 2000 paddy fields were randomly selected from the study area. The sampling scheme was designed to ensure broad spatial coverage across the study region, allowing these fields to serve as representative units for subsequent pseudo-label reconstruction.

#### 2.2.4 Data alignment and sequence construction

To fuse multimodal data with different temporal resolutions, a daily sequence was constructed for each field-year from planting to harvest. Meteorological variables were available at daily resolution and were directly aligned to the daily time axis. Sentinel-2 vegetation indices were temporally irregular because of revisit intervals and cloud contamination, and were therefore assigned only to their actual acquisition dates. For dates without valid satellite observations, the corresponding vegetation-index inputs were filled with zeros and paired with a binary mask indicating whether a valid remote-sensing observation was available on that day (1 = valid observation, 0 = missing observation). This alignment process resulted in a unified tensor structure containing continuous meteorological drivers and discontinuous remote sensing features, serving as the direct input for the LSTM framework.

### 2.3 Construction of synthetic dataset via crop modelling

#### 2.3.1 The CERES-rice model

The CERES-Rice model within the DSSAT cropping system was used to simulate rice phenology, growth, biomass accumulation, and N uptake under diverse environmental and management conditions. CERES-Rice represents crop development through daily thermal time accumulation and photoperiod response, and dynamically simulates leaf and tiller production, canopy growth, soil water and N cycling, and biomass partitioning throughout vegetative and reproductive phases [19]. Its process-based structure allows explicit integration of genotype, weather, soil properties, and management practices into a unified simulation framework.

Prior to the regional multi-scenario simulations, the DSSAT model (CERES-Rice module) was calibrated and validated to reproduce local rice phenology and growth dynamics. Genetic coefficients were adjusted to match observed phenology, biomass, and yield derived from our field experiments. Details of the calibration procedure, calibration-validation data partitioning and statistical evaluation metrics are provided in *Supplementary Materials*. The evaluation results demonstrated that the calibrated model performed robustly in simulating crop development across different N treatments with simulated values showing high consistency with independent field measurements (Fig. S2–6).

#### 2.3.2 Building the simulation scenario library

To construct a comprehensive source domain dataset capturing spatiotemporal variability, simulations were conducted across the rice planting area of Xinghua City for the 2020-2024 growing seasons. The study region was rasterized into simulation units at a 10 km × 10 km resolution, where the DSSAT model was initialized with spatially explicit environmental inputs for each grid cell. Soil physical and chemical parameters were extracted from the SoilGrids database (ISRIC) to define the specific soil profiles, while daily meteorological drivers were derived from the interpolated station data (as described in Section 2.2). Within each spatiotemporal unit (Grid×Year), a scenario library was simulated consisting of 700 management combinations, comprising 14 N rates, 10 transplanting dates, and 5 fertilizer split ratios (Table 2). This process generated a massive pool of potential growth trajectories tailored to the local soil and weather conditions of each grid.

**Table 2:** Experimental factors and levels used for generating DSSAT multi-scenario pseudo-labels.

Factor	Levels	Description
Study period	5 years	2020-2024 rice-growing seasons
Total nitrogen rate (N_total)	14 levels	100-360 kg ha^−1^ (20 kg increments)
Nitrogen application ratio	5 schemes	R1: 3:3:2:2
		R2: 3:2:3:2
		R3: 4:3:2:1
		R4: 3:2:5:0
		R5: 4:2:2:2
Transplanting dates	10 dates	June 1 to 30 (3-day interval)
Number of combinations	700	Scenarios per field-year

Note: Fertilizer split ratios indicate the proportion of total nitrogen applied at four fixed growth stages: basal application, tillering, jointing, and panicle initiation.

#### 2.3.3 Derivation of fPAR as the bridging variable

To link the physiological outputs of DSSAT with optical satellite observations, the fraction of absorbed photosynthetically active radiation (fPAR) was utilized as a common proxy variable. Since fPAR is not a direct output of either system, it was derived and normalized as follows:

1 Simulated fPAR from DSSAT

The DSSAT model provides the daily leaf area index (LAI) outputs. Based on the Beer-Lambert law [20,21], simulated ${fPAR}_{sim}$ was calculated as:

(1) $$\begin{array}{c} {fPAR}_{sim}=1-e^{\left(-k\cdot {LAI}_{sim}\right)} \end{array}$$

where *k* is the canopy extinction coefficient, set to 0.6 for rice.

2. Observed fPAR from Sentinel-2

For satellite observations, ${fPAR}_{obs}$ was derived from the NDVI using a linear scaling model based on the spectral statistics [22,23]:

(2) $$\begin{array}{c} {fPAR}_{obs}={fPAR}_{\min}+\frac{\left(NDVI-{NDVI}_{\min}\right)}{{NDVI}_{\max}-{NDVI}_{\min}}\;\cdot \left({fPAR}_{\max}-{fPAR}_{\min}\right) \end{array}$$

where ${NDVI}_{\min}$ and ${NDVI}_{\max}$ represent the NDVI values for bare soil and closed canopy, respectively, while ${fPAR}_{\min}$ (0.001) and ${fPAR}_{\max}$ (0.95) are the corresponding theoretical bounds. Values exceeding these bounds were clamped to the valid range. Finally, to ensure comparability of curve shapes and eliminate absolute magnitude discrepancies, both time series were normalized to the [0, 1] range using min-max scaling.

#### 2.3.4 Scenario matching strategy and pseudo-label dataset construction

To reconstruct physiologically consistent pseudo-labels for the 2000 representative fields, a multi-metric matching strategy was employed to link the field-specific remote sensing observations with the DSSAT simulation library. First, for each real-world field, the specific scenario library (comprising 700 management combinations) was retrieved based on the field's spatial coordinates (assigning it to the corresponding 10 km × 10 km grid) and the specific growing season (year). This ensured that the candidate simulation trajectories shared the identical baseline meteorological and soil conditions as the target field. Second, after temporal alignment between observed and simulated fPAR series, the most probable growth trajectory was identified from the simulation library using a multi-metric matching approach. The similarity between the observed satellite series (${fPAR}_{obs}$) and each simulated scenario (${fPAR}_{sim}$) was evaluated using a composite score incorporating three metrics: (a) Pearson correlation (*r*): which captures the linear correlation of growth trends. (b) cosine similarity (Cosine): which measures the consistency of the curve shapes [24]. (c) dynamic time warping (DTW): which quantifies temporal alignment while accommodating phenological shifts and data gaps [25]. The comprehensive matching score was defined as:

(3) $$\begin{array}{c} score=wr\cdot r+wc\cdot Cosine-wd\cdot \frac{DTW}{{DTW}_{\max}} \end{array}$$

where wr, wc, and wd are weighting factors balancing the contributions of trend, shape, and temporal alignment. ${DTW}_{\max}$ is the maximum DTW distance across all candidate scenarios for that field and was used to normalize the DTW term. In this study, wr = 0.4, wc = 0.4, and wd = 0.2 were used, ensuring that correlation, cosine similarity, and DTW distance contribute comparably to the composite score. A sensitivity analysis of alternative weighting schemes is provided in Fig. S10 in the *Supplementary Materials*.

**Fig. 2:**
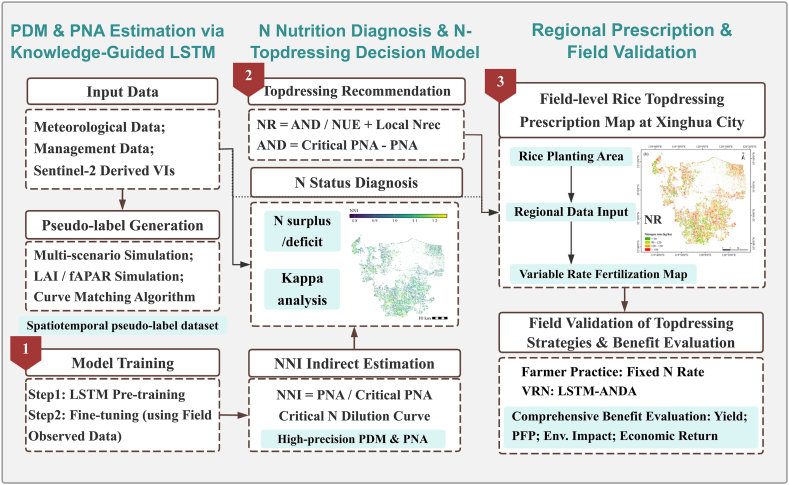
fPAR dynamics derived from DSSAT-simulated LAI for different management scenarios within the selected field. Black points represent satellite-retrieved fPAR observations for the field. The red curve corresponds to the optimally matched trajectory.

Based on this evaluation, the DSSAT simulation scenario corresponding to the highest matching score was selected as the best-fitting trajectory (Eq. (3)), as illustrated by an example field in Fig. 2. The daily PDM and PNA values from the selected trajectory were then extracted as DSSAT-derived pseudo-labels for the corresponding field. The final reconstructed dataset therefore consisted of daily input sequences and matched PDM-PNA pseudo-label trajectories for all 2000 representative fields. The independent variables were organized into a daily tensor aligning meteorological drivers, spectral features, and management data. Specifically, the meteorological variables included daily maximum air temperature (MAX), daily minimum air temperature (MIN), average daily air temperature (TAV), daily solar radiation (SAR), daily precipitation (RAIN), cumulative solar radiation (SAR_CUM), cumulative precipitation (RAIN_CUM), and accumulated growing degree days (AGDD). The management variables comprised basal nitrogen application (BN) and days after transplanting (DAT). The spectral variables included NDVI, NDRE, GNDVI, RESAVI, and CIRE. The output variables were the daily PDM and PNA trajectories extracted from the optimally matched DSSAT scenarios.

### 2.4 Deep learning model and transfer learning strategy

#### 2.4.1 LSTM network architecture

To capture the temporal dynamics and cumulative effects of environmental factors on rice growth, LSTM networks were adopted due to their ability to model long-term temporal dependencies through gated memory mechanisms, which is essential for representing cumulative crop growth processes [9,10,26]. The model architecture consists of an input layer, two stacked LSTM layers with 128 hidden units each, and a fully connected output layer. To handle irregular temporal observations and missing reference values, a mask-aware loss function was implemented during training. Specifically, the mean-squared error (MSE) was computed only on valid time steps, ensuring that zero-padded positions and missing labels did not bias the gradient estimation. The optimization was performed using the Adam optimizer with a learning rate of 0.001 and a batch size of 64, and the network was trained for 50 epochs. At each time step t, the model only uses information that would be available up to time t (i.e., historical meteorological variables, past satellite observations, and management records), without incorporating any future information, ensuring strict causal consistency for in-season diagnosis.

#### 2.4.2 Pretraining using model-generated growth trajectories

The LSTM model was pretrained using the reconstructed paired dataset described in Section 2.3.4, where daily meteorological, spectral, and management inputs were aligned with DSSAT-derived PDM and PNA trajectories serving as pseudo-labels. The simulated dataset was split by year, with one growing season held out as a pseudo-label validation set and the others used for training. Feature scalers were fitted only on the training subset to avoid information leakage.

#### 2.4.3 Transfer learning and model fine-tuning

To transfer the pretrained LSTM to field conditions, the model was fine-tuned through a staged adaptation process using real field measurements. During this procedure, the lower recurrent layers were retained from the pretraining phase and kept fixed, as they encoded generalizable temporal patterns of rice growth. The upper recurrent layer and the final fully connected layer were set to be trainable to accommodate field-specific dynamics.

To ensure consistency between the simulation and real-world domains, the feature and target scalers derived from the pretraining phase were directly applied to the field observations. The model was then fine-tuned with a small learning rate under the same mask-aware loss function. The fine-tuning hyperparameters were selected under the field-level five-fold cross-validation framework described in Section 2.5. The main hyperparameters considered included the learning rate and number of training epochs, and model selection was based on the minimum validation RMSE averaged across folds. Based on this procedure, the final prediction model was retrained using 100% of the available field observations with the selected hyperparameters (learning rate = 0.0001, epochs = 30). Other architectural and pretraining settings, including the number of LSTM layers, hidden units, batch size, and pretraining epochs, were fixed before field fine-tuning and are summarized in Table S2.

### 2.5 Model implementation and evaluation strategy

To evaluate the performance of the proposed framework, the experiment was organized into two major stages: (i) simulation-based pretraining and (ii) field-based transfer learning.

In the first stage, a leave-one-year-out cross-validation (LOYO-CV) strategy was applied to the 2020-2024 simulated dataset. In each iteration, four years were used for training and the held-out year served as an independent pseudo-label validation set. Performance on this pseudo-label validation set was quantified using the coefficient of determination (R^2^), root mean squared error (RMSE), and relative RMSE (RRMSE) (Eqs. (4)–(6)), ensuring the model had converted to a stable physiological representation before transfer learning.

(4) $$\begin{array}{c} R^{2}=\frac{\sum_{i=1}^{n}{\left(O_{i}-{\bar{O}}_{i}\right)}^{2}{\left(P_{i}-{\bar{P}}_{i}\right)}^{2}}{\sum_{i=1}^{n}{\left(O_{i}-{\bar{O}}_{i}\right)}^{2}\sum_{i=1}^{n}{\left(P_{i}-{\bar{P}}_{i}\right)}^{2}}\; \end{array}$$

(5) $$\begin{array}{c} RMSE=\sqrt{\frac{1}{n}\times \sum_{i=1}^{n}{\left(P_{i}–O_{i}\right)}^{2}} \end{array}$$

(6) $$\begin{array}{c} RRMSE=\frac{RMSE}{sd}\times 100 \end{array}$$

where n is the sample size, $O_{i}$ and $P_{i}$ denote the observed and predicted values, and ${\bar{O}}_{i}$ and ${\bar{P}}_{i}$ represent their corresponding means.

In the second stage, the pretrained model was adapted to real field conditions using observed PDM and PNA measurements collected in Exp 2. To rigorously assess model robustness and select optimal hyperparameters, a field-level five-fold cross-validation (5-fold CV) strategy was implemented. The real-world dataset was partitioned into five folds based on unique field IDs. In each iteration, four folds were used for fine-tuning, and the remaining fold served as the validation set. This ensured that observations from the same field were never split between training and validation, preventing spatial information leakage. The evaluation metrics reported in this study represent the aggregated performance across all five validation folds. Performance evaluation throughout the transfer learning stage relied on R^2^, RMSE, and nRMSE. Their calculation formulas are presented in Eqs. (4)–(6). To improve model interpretability, a post-hoc perturbation-based sensitivity analysis was conducted after model evaluation. Specifically, a fixed positive perturbation (+0.5 in the standardized input space) was applied to each input variable individually while all remaining inputs were kept unchanged, and the resulting changes in model predictions were quantified. The averaged perturbation-induced changes at valid time steps were used to examine how model outputs responded to different spectral, temporal, meteorological, and management variables.

To benchmark the LSTM against conventional machine learning approaches, Random Forest (RF), XGBoost, Support Vector Machine (SVM), Neural Network (NNET), and Partial Least Squares regression (PLS) were trained using the same input features and evaluated under the same field-level 5-fold CV protocol as the fine-tuned LSTM. These models served as non-sequential baselines, with cumulative agro-meteorological features incorporated to provide temporal context. For all baseline models, hyperparameters were tuned using grid search implemented in the R caret package, with the optimal settings selected by minimizing the RMSE averaged across validation folds; the detailed tuning process and optimal parameters are illustrated in Fig. S7 and Table S1.

### 2.6 Nitrogen nutrition diagnosis, variable-rate recommendation, and benefit assessment

Based on the daily trajectories of PDM and PNA reconstructed by the model, rice N status was quantified using the NNI. NNI represents the relative N sufficiency of the crop and is defined as the ratio of the actual PNA to its critical PNA required for maximum growth. The critical N concentration ($N_{c}$) was obtained from a region-specific critical N dilution curve for rice developed by Fu, Zhang, Zhang, Zhang, Cao, Tian, Zhu, Cao and Liu [27]. This dilution curve describes the decline of $N_{c}$ as PDM increases and is expressed as:

(7) $$\begin{array}{c} N_{c}=3.57\times {PDM}^{-0.31} \end{array}$$

Accordingly, NNI was calculated as:

(8) $$\begin{array}{c} NNI=\frac{PNA}{PDM\times N_{c}} \end{array}$$

where $PDM\times N_{c}$ represents the critical PNA.

Based on the critical N dilution curve (Eq. (7)), the AND was further quantified to characterize the cumulative N deficiency of the crop relative to its critical N demand. AND was calculated as:

(9) $$\begin{array}{c} AND=PDM*N_{c}-PNA\; \end{array}$$

Using these formulations, NNI was estimated for each field and date covered by the fine-tuned LSTM model output. The accuracy of NNI and AND estimation was evaluated using an independent ground-validation dataset described in Section 2.1 (Exp 2). Prediction accuracy was quantified using R^2^ and RMSE by comparing predicted and observed NNI values. Categorical N status was assessed by assigning each sample to one of three N nutritional classes—N-deficient, N-optimal, and N-excessive—based on the established diagnostic interval for rice NNI (0.97-1.16) reported by Yang, Jiang, Fu, Wang, Cao, Tian, Zhu, Cao and Liu [28]. Cohen's Kappa coefficient was calculated to quantify the agreement between model-derived and observed classifications. To guide variable-rate N management, the N requirement (NR) was computed by integrating the AND into a practical fertilization rule:

(10) $$\begin{array}{c} NR=N_{local}+\frac{AND}{NUE} \end{array}$$

where $N_{local}$ denotes the locally recommended topdressing rate based on regional agronomic guidelines, and NUE represents the N use efficiency. Following previous studies and regional fertilization practices [29], $N_{local}$ and NUE were set to 135 kg ha^−1^ and 0.5, respectively.

To explicitly quantify the agronomic and economic benefits of this variable-rate strategy compared to traditional farmers' practices (FP), two key performance indicators were introduced: partial factor productivity of N (NPFP) and net profit (NP). NPFP reflects the grain yield produced per unit of N applied and is calculated as:

(11) $$\begin{array}{c} NPFP\;\left(kg\cdot {kg}^{-1}\right)=\frac{GY}{N_{total}} \end{array}$$

where GY is the grain yield and $N_{total}$ is the total applied N. NP assesses economic feasibility by considering fertilizer costs and grain income:

(12) $$\begin{array}{c} NP\;\left(CNY\cdot {ha}^{-1}\right)=GY\times P_{grain}-N_{total}\times P_{fert} \end{array}$$

where $P_{grain}$ and $P_{fert}$ represent the market prices of rice grain (2.80 $CNY\cdot {kg}^{-1}$) and N fertilizer (5.53 $CNY\cdot {kg}^{-1}$) respectively.

Finally, the proposed framework was extended to regional-scale N diagnosis and recommendation mapping. Model-estimated PDM and PNA were first generated for all rice fields across the Xinghua rice-growing region on 31 July 2024, corresponding to the jointing stage. These spatially estimates were then converted into NNI and NR maps using Eqs. (7), (8), (9), (10), enabling field-specific N status assessment and variable-rate fertilization planning at the landscape scale.

## 3 Results

### 3.1 Performance evaluation of the physiology-informed LSTM framework

#### 3.1.1 Pseudo-label reliability and mechanistic knowledge internalization

To evaluate the reliability of CERES-Rice-derived pseudo-labels, time-series curve matching was conducted between simulated and observed crop growth trajectories. Overall, Pearson correlation coefficients and cosine similarity values remained consistently high across years, while DTW distances were relatively low, indicating good temporal alignment (Table 3).

**Table 3:** Summary statistics of time-series curve matching metrics for best-matched simulated growth trajectories against observations.

Year	Pearson correlation (r)		Cosine similarity		DTW distance	
	Mean	SD	Mean	SD	Median	IQR
2020	0.94	0.14	0.98	0.04	0.70	0.47-1.01
2021	0.87	0.15	0.97	0.03	1.26	0.71-1.78
2022	0.94	0.13	0.97	0.05	0.64	0.44-0.92
2023	0.95	0.13	0.98	0.04	0.56	0.25-0.80
2024	0.78	0.28	0.99	0.02	0.68	0.37-1.06
All years	0.90	0.19	0.98	0.04	0.70	0.44-1.19

Note: Pearson correlation coefficient (r) and cosine similarity are reported as mean and standard deviation (SD), summarizing the consistency of temporal patterns. Dynamic Time Warping (DTW) distance is reported as the median with interquartile range (IQR; 25th-75th percentile) due to its skewed distribution. For each year, statistics were calculated based on the best-matched simulated trajectory for each field, selected using the composite similarity score.

The three representative samples further illustrate the dynamic consistency between DSSAT-simulated and LSTM-predicted growth trajectories (Fig. S8). Consistent with the high temporal similarity metrics reported in Table 3, the LSTM successfully reproduced stage-specific growth dynamics, including rapid biomass accumulation during vegetative stages and the subsequent stabilization toward maturity. Across all examples, the predicted curves closely tracked the simulated trajectories without noticeable phase shifts or structural distortions.

**Fig. 3:**
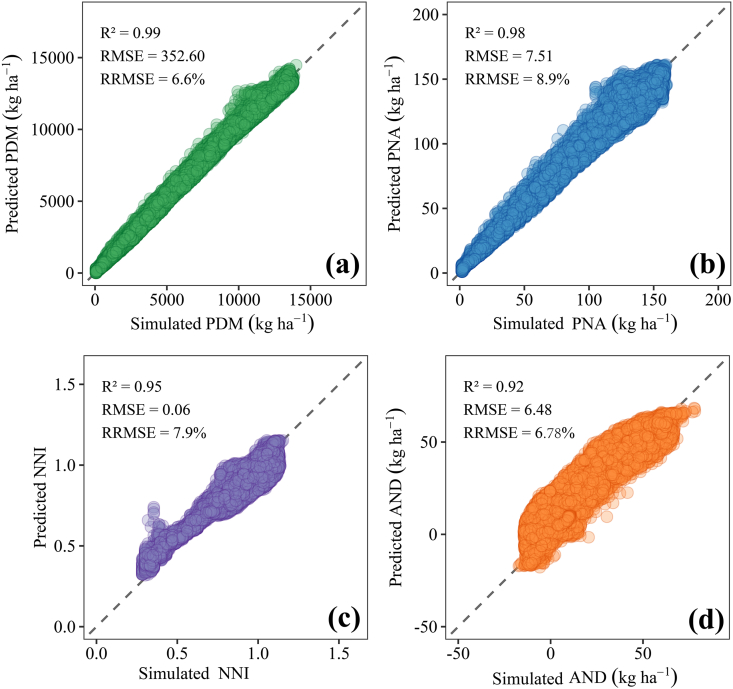
Scatter plots showing the agreement between pseudo-label values derived from the matched DSSAT trajectories and LSTM predictions on the synthetic hold-out test set for (a) plant dry matter (PDM), (b) plant nitrogen accumulation (PNA), (c) nitrogen nutrition index (NNI), and (d) accumulated nitrogen deficiency (AND). The 1:1 dashed line indicates perfect agreement between predicted and reference values.

To assess whether the LSTM network successfully internalized the growth mechanisms embedded in the biophysical model, its performance on the synthetic hold-out test set was first evaluated (Fig. 3).

The scatter plots show that predictions for both PDM and PNA were tightly distributed around the 1:1 line, indicating that the pretrained network effectively reproduced the pseudo-label trajectories generated from the matched DSSAT scenarios. The model achieved near-perfect agreement with the pseudo-labels for PDM and PNA, with R^2^ values of 0.99 and 0.98, RMSE values of 352.6 kg ha^−1^ and 7.51 kg ha^−1^, and RRMSE values of 6.6% and 8.9%, respectively, indicating that the model effectively learned the deterministic patterns embedded in the pseudo-label space during pretraining. Furthermore, derived N diagnostic indicators were also accurately reproduced. The predicted NNI and AND achieved R^2^ values of 0.95 and 0.92, with RMSE values of 0.06 and 6.48 kg ha^−1^, and RRMSE values of 7.9% and 6.78%, respectively.

#### 3.1.2 Field adaptation and comparative model performance

**Fig. 4:**
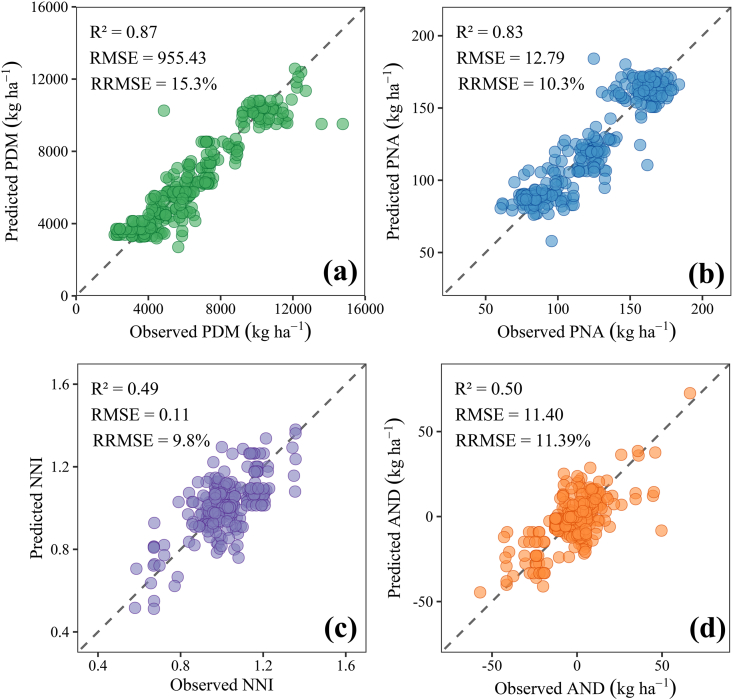
Scatter plots of observed versus predicted values from the field-level five-fold cross-validation of the fine-tuned LSTM model for (a) plant dry matter (PDM), (b) plant nitrogen accumulation (PNA), (c) nitrogen nutrition index (NNI), and (d) accumulated nitrogen deficiency (AND). Each point represents a field observation in the validation folds. The dashed 1:1 line denotes perfect prediction.

To rigorously assess the generalization capability of the proposed framework, model performance was evaluated using 5-fold cross-validation on the field observation dataset (Fig. 4). The scatter plots indicate that predicted and observed values for PDM and PNA were generally well aligned along the 1:1 line, confirming that the fine-tuned LSTM retained robust predictive capability under real-world conditions. In contrast, the scatter distributions for NNI and AND were visibly more dispersed, suggesting that uncertainty increased when these diagnostic indicators were derived from the estimated biophysical variables. For PDM, the model achieved an R^2^ of 0.87, with an RMSE of 955.43 kg ha^−1^ and an RRMSE of 15.3%. For PNA, the predictions yielded an R^2^ of 0.83, an RMSE of 12.79 kg ha^−1^, and an RRMSE of 10.3%. Regarding N diagnostic indicators, the NNI and AND achieved R^2^ values of 0.49 and 0.50, respectively, with RMSE values of 0.11 and 11.40 kg ha^−1^, and RRMSE values of 9.8% and 11.39%.

To further evaluate the advantage of the temporal modeling architecture, the fine-tuned LSTM was compared with several widely used machine-learning algorithms, including support vector machine (SVM), random forest (RF), partial least squares (PLS), extreme gradient boosting (XGB), and a feedforward neural network (NNET) (Table S3). For PDM, the LSTM achieved the best overall performance, with the lowest RMSE (955.43 kg ha^−1^) and RRMSE (15.30%), and the highest R^2^ (0.87). In comparison, the other models showed higher prediction errors, with RRMSE values ranging from 16.42% (SVM) to 18.13% (NNET), and slightly lower R^2^ values (0.83-0.86). A similar pattern was observed for PNA. The LSTM outperformed all benchmark models, achieving an RMSE of 12.79 kg ha^−1^, an R^2^ of 0.83, and the lowest RRMSE (10.30%). In contrast, the competing algorithms exhibited higher RRMSE values between 12.54% and 13.15%, accompanied by lower R^2^ values (0.80-0.81). Statistical significance analysis based on the five-fold cross-validation results further confirmed significant overall differences among models for all four metrics in both PDM and PNA, as indicated by Friedman tests (Table S4). Post-hoc paired Wilcoxon signed-rank tests with Holm correction showed that the LSTM generally achieved better average performance compared with the baseline models, although not all pairwise comparisons remained statistically significant after correction.

#### 3.1.3 Global sensitivity analysis of model features

**Fig. 5:**
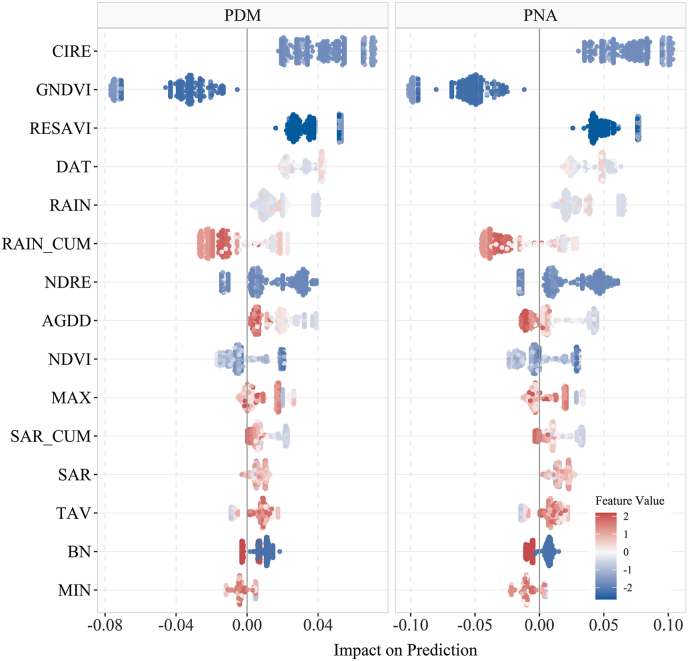
Global sensitivity analysis of input features for predicting rice PDM and PNA.

To understand the driving factors behind the model's predictions, a post-hoc global sensitivity analysis was conducted (Fig. 5). The analysis showed that the PI-LSTM predictions responded to a combination of spectral, temporal, and meteorological inputs rather than relying on a single data modality. Specifically, larger perturbation-induced responses were observed for red-edge and green-based spectral indices (CIRE, GNDVI, RESAVI), temporal markers (DAT), and hydrological factors (RAIN, RAIN_CUM). By contrast, static management variables such as basal N rate generally induced smaller prediction changes. These results suggest that the model primarily captured dynamic crop and environmental signals when estimating PDM and PNA.

### 3.2 Evaluation of N status diagnosis and regional variable-rate prescription

**Fig. 6:**
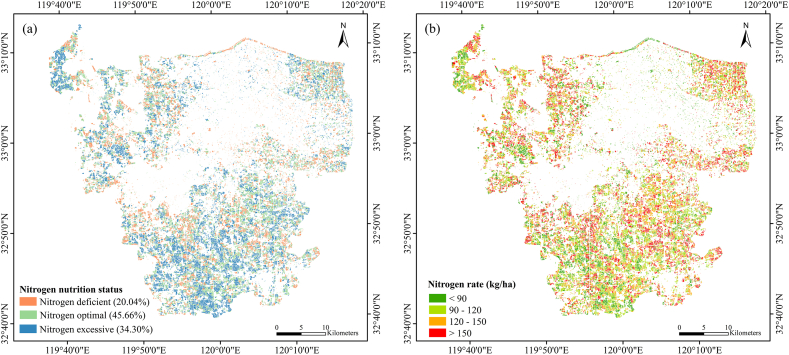
Regional maps of rice nitrogen status and fertilizer requirement across the Xinghua rice-growing area on 31 July 2024. (a) Field-level NNI diagnostic classes, and (b) corresponding nitrogen requirement (NR, kg·ha^−1^) derived from estimated AND.

The diagnostic performance was evaluated by categorizing field samples into three N status classes (Deficient, Optimal, Excessive). The fine-tuned LSTM achieved an overall accuracy (OA) of 67.3% with a Kappa coefficient of 0.51. To demonstrate the operational capability of the framework, the model was applied to the entire Xinghua rice-growing region. By integrating time-series Sentinel-2 observations, meteorological data, and management information, the spatial distributions of PDM and PNA were estimated for 31 July 2024 (Fig. S9). Using these spatially explicit PDM and PNA estimates as foundational variables, the regional NNI and subsequent NR rates were calculated. Subsequently, the regional N status in Xinghua was characterized using the derived NNI diagnostic map (Fig. 6a). Based on the optimal NNI range (0.97-1.16), the region exhibited clear spatial heterogeneity: while 45.66% of fields were N-optimal, 20.04% were identified as N-deficient, and notably, 34.30% were N-excessive. Building upon the NNI diagnosis, field-level N fertilizer recommendations were further derived by adjusting the $N_{local}$ according to the AND. At the regional scale, the median and mean N requirements were 127.70 and 128.29 kg ha^−1^, respectively, with an interquartile range of 104.11-154.00 kg ha^−1^, underscoring the inadequacy of uniform fertilization strategies for addressing spatially heterogeneous crop N demand (Fig. 6b).

### 3.3 Variable-rate fertilization strategy and on-farm verification

To validate the agronomic and economic benefits of the proposed framework, on-farm comparative experiments were conducted across seven sites during the 2024 and 2025 growing seasons (Table 4).

**Table 4:** Comparisons of grain yield (GY), nitrogen partial factor productivity (NPFP), and net profit (NP) between Farmers’ Practice (FP) and the proposed ANDA across different experimental sites.

Year	Site	BBCH	Treatment	NR (kg·ha^−1^)	GY (t·ha^−1^)	NPFP (kg·kg^−1^)	NP (CNY·ha^−1^)
2024	LC_E	27	FP	342	8.22 ab	24.0b	15065 ab
			ANDA	291	8.39a	28.8a	15838a
	DY	30	FP	333	11.47a	34.5b	24229a
			ANDA	286	11.24a	39.3a	23838a
	ZZ	32	FP	330	9.03a	27.4b	17415b
			ANDA	285	9.64a	33.8a	19357 ab
	LC_L	34	FP	337	10.87a	32.3b	22526a
			ANDA	296	10.66a	36.0a	22150a
2025	XW	34	FP	330	10.80a	32.7a	22362a
			ANDA	322	11.03a	34.3a	23050a
	DY	32	FP	300	11.47a	38.2a	24404a
			ANDA	286	11.44a	40.0a	24397a
	LS	34	FP	360	9.87b	27.4b	19592b
			ANDA	331	11.18a	33.8a	23421a

Note: LC_E and LC_L represent topdressing experiments conducted in LC Farm at different phenological stages. BBCH values indicate the growth stage at topdressing; values sharing the same letter indicate no significant difference.

Compared with the Farmers' Practice (FP), the accumulated nitrogen deficiency-based algorithm (ANDA) consistently optimized N inputs while maintaining or increasing grain yield. Specifically, ANDA reduced the total N application rate by an average of 13.4% compared to FP. Despite the reduced N input, rice grain yield under ANDA was generally maintained across sites, with a significant yield increase of 13.3% observed at the LS site in 2025. In terms of nitrogen use efficiency, ANDA generally improved NPFP across sites, although the improvement was not statistically significant at all locations. Significant increases in NPFP were observed at LC_E, DY (2024), ZZ, LC_L, and LS, while changes at XW and DY (2025) were not significant. For economic performance, ANDA achieved numerically higher NP in most trials; however, these increases were generally not statistically significant. A significant increase in NP was observed only at the LS site in 2025, where NP increased by 19.5% relative to FP. These results demonstrate that the physiology-informed LSTM framework can effectively guide regional variable-rate fertilization by reducing N inputs and improving N-use efficiency, while generally maintaining yield and avoiding short-term economic penalties.

## 4 Discussion

### 4.1 Mechanisms underlying the enhanced performance of the physiology-informed LSTM framework

The proposed PI-LSTM framework demonstrates improved performance in estimating rice PDM and PNA by effectively bridging the gap between mechanistic crop modeling (DSSAT) and remote sensing (Sentinel-2). Traditional data-driven approaches often treat satellite observations as independent, static snapshots, failing to capture the cumulative nature of biomass accumulation and N uptake. In contrast, the temporal matching results (Table 3 and Fig. 3) highlight the LSTM's inherent advantage: its memory cells can track the sequential progression of crop phenology, effectively mimicking the temporal dynamics modeled by DSSAT [30]. However, training such complex sequence models typically requires prohibitively large datasets, which are unattainable through conventional destructive field sampling. The proposed framework provides a potential solution to this bottleneck by using DSSAT-derived pseudo-labels as a source domain for pretraining. By pre-training on massive DSSAT-derived pseudo-labels, the deep learning model not only satisfies its extensive data requirements but also internalizes general physiological constraints. This pre-training ensures that the model learns the biophysical boundaries of crop growth, preventing the physiologically implausible fluctuations often seen in purely data-driven approaches when faced with missing or noisy satellite data [6,9]. It should be noted, however, that the near-perfect performance achieved during pseudo-label validation should be interpreted cautiously. Because this stage evaluates the model's ability to reproduce DSSAT-derived pseudo-labels in a physiologically constrained simulation space, these high R^2^ values mainly indicate successful internalization of mechanistic growth dynamics rather than true predictive performance under real-world field conditions. In real production systems, unmodeled disturbances such as disease or pest pressure and lodging may introduce biases into the pseudo-labels. Field-based fine-tuning with multi-year observations was therefore used to adapt the pretrained model to real production conditions.

When fine-tuned and applied to real-world field conditions, the PI-LSTM maintained robust predictive accuracy, consistently outperforming widely used machine-learning algorithms (e.g., RF, SVM, and XGBoost). This comparative advantage primarily stems from the handling of temporal dependencies. Static models like RF treat multi-temporal observations as isolated features, limiting their ability to capture the historical legacy of crop stress. Additionally, tree-based models typically struggle to extrapolate beyond the distribution of their training data. The LSTM, however, recursively updates the crop state, allowing it to better capture the complex, non-linear trajectories of crop physiological status under fluctuating growing conditions [31]. Furthermore, the global sensitivity analysis confirms that the fine-tuned framework explicitly fuses real-time canopy diagnosis (via spectral signals) with phenological baselines (via DAT) and environmental constraints (via precipitation). This robust agronomic logic effectively mitigates the saturation issues often encountered by traditional indices in dense rice canopies [32]. Although this study focused on rice, the general PI-LSTM logic may be transferable to other staple crops, but its practical applicability would require further validation and crop-specific recalibration of phenological, remote-sensing, and N diagnostic relationships.

### 4.2 Agronomic and economic assessment of the proposed framework

Building upon accurate PDM and PNA estimations, this study established an indirect NNI diagnostic framework. Although error propagation from intermediate variables is inevitable, the fine-tuned model achieved an OA of 67.3% and a Kappa coefficient of 0.51 for N status classification, indicating moderate diagnostic agreement [33]. The moderate diagnostic performance may be partly attributed to the use of a general critical N dilution curve developed by Fu et al. [27] across all fields in this study. Although this curve provides a practical basis for regional N diagnosis, its parameters may not fully capture variations in cultivar, site-specific soil fertility, management practices, and year-to-year growing conditions. Such uncertainty can affect the estimation of critical PNA and consequently influence NNI-based classification. The regional diagnosis suggests that a substantial proportion of fields may receive more N than required, consistent with risk-averse fertilization practices commonly observed in intensive rice production. Such risk-averse fertilization may lead to luxury N consumption and increased environmental risks [34]. By translating model-derived AND into field-specific topdressing rates, the proposed framework offers a practical way to better match N supply with crop demand.

The agronomic and economic viability of this framework was further evaluated through on-farm comparative experiments across seven sites during the 2024 and 2025 growing seasons (Table 4). The capacity of ANDA to maintain or, in some cases, increase grain yield while reducing fertilizer input highlights the importance of optimizing the spatial distribution of N rather than merely increasing total rates. Correspondingly, NPFP was generally improved under ANDA, although the improvement was not statistically significant at all sites. However, these agronomic gains did not always translate into statistically significant short-term economic benefits. This discrepancy likely reflects the relatively small monetary contribution of fertilizer-cost savings compared with yield-driven revenue variation, as well as site-specific differences in yield formation, management costs, and grain prices. The significant NP improvement observed at the LS site in 2025 further suggests that the economic advantage becomes more detectable when fertilizer reduction is coupled with a yield increase. Therefore, the value of ANDA should be interpreted not only in terms of immediate net profit, but also through its potential to improve N-use efficiency and reduce environmental N losses.

Taken together, these findings indicate that the PI-LSTM framework has potential as a scalable decision-support tool for regional precision N management. Its primary value lies in translating PI-LSTM-derived crop N status into field-differentiated topdressing recommendations through the ANDA strategy, thereby improving N-use efficiency and maintaining yield stability while potentially creating site-specific opportunities for economic gains [4,35,36]. By distinguishing N-deficient fields from those already sufficient or excessive, it helps correct the “insurance-driven” over-fertilization commonly observed in local practices.

### 4.3 Limitations

Despite the robust agronomic outcomes validated across the 2024 and 2025 seasons, several limitations within the proposed PI-LSTM framework remain. A primary limitation of the proposed PI-LSTM framework lies in the pseudo-label generation process. Because DSSAT simulates crop growth under defined environmental and nutrient constraints, the reconstructed trajectories represent a physiologically attainable growth state rather than the full complexity of actual field conditions. Although field-based fine-tuning helped adapt the pretrained model to real observations, these biases may not be completely eliminated [37].

Another limitation arises from the use of a fixed regional critical nitrogen dilution curve. Although the curve provides a practical basis for N status diagnosis, its parameters may vary across genotypes, environments, and management conditions [27], thereby introducing uncertainty into the estimation of critical PNA, NNI, and AND. Future work should develop adaptive strategies to dynamically calibrate dilution-curve parameters under varying production scenarios. In addition, management inputs were simplified at the regional scale, which may not fully capture field-level differences in planting dates, fertilization timing, irrigation, or farmer practices. Therefore, although the proposed framework showed promising within-region performance, applications to new rice cultivars or substantially different growing regions may require local recalibration or lightweight fine-tuning to reduce genotype-environment-management domain shifts.

Finally, the current framework relies on optical and meteorological data but overlooks subsurface hydrology. Future iterations will aim to integrate spatially explicit soil moisture constraints to better regulate N leaching and root uptake dynamics, further refining the model's generalization capabilities under complex environmental conditions.

## 5 Conclusion

This study developed a physiology-informed LSTM framework for regional-scale rice nitrogen management by integrating DSSAT-derived physiological knowledge with Sentinel-2 time-series observations and meteorological data. The proposed framework provides a feasible strategy to alleviate the limitation of sparse field observations in remote sensing-based nitrogen diagnosis and to support the estimation of crop growth and nitrogen status indicators. After field fine-tuning, the model achieved R^2^ values of 0.87 and 0.83 for PDM and PNA, respectively, and the derived NNI classification reached an overall accuracy of 67.3%. Field evaluation further demonstrated that the framework can support reliable nitrogen diagnosis and field-specific topdressing decisions under heterogeneous production conditions. Compared with farmers’ practices, the ANDA strategy reduced N input by 13.4% and improved nitrogen partial factor productivity by 18.6% while maintaining grain yield. However, statistically significant increases in net profit were observed only under specific site-year conditions, indicating that short-term economic benefits were more dependent on local yield responses and production conditions. Overall, the proposed framework provides practical support for field-level nitrogen management, offers region-specific information for nutrient management planning, and may serve as a component of operational precision-agriculture systems. Nevertheless, its performance still depends on the fidelity of DSSAT simulations and the current framework does not explicitly account for acute stress events or soil hydrological processes, which should be addressed in future work.

## CRediT authorship contribution statement

Jinpeng Yang: Writing – review & editing, Writing – original draft, Methodology. Zhaopeng Fu: Writing – review & editing. Ke Zhang: Investigation, Data curation. Bohuai Shi: Investigation. Jiang Wang: Software. Weizhe Zhao: Software. Qiang Cao: Resources. Yongchao Tian: Validation. Yan Zhu: Funding acquisition. Weixing Cao: Validation, Resources, Funding acquisition, Data curation. Xiaojun Liu: Supervision, Funding acquisition.

## Funding

This work was supported by the Agricultural Science and Technology Innovation Program of Shanghai (No. I2023005), the National Key Research and Development Program of China (No. 2023YFD1701000), the Frontier Technologies R&D Program of Jiangsu (No. BF2025310), the National Postdoctoral Program for Innovative Talents (No. GZC20252646), and the Jiangsu Funding Program for Excellent Postdoctoral Talent (No. 2025ZB861).

## Declaration of competing interest

The authors declare that they have no known competing financial interests or personal relationships that could have appeared to influence the work reported in this paper.

## Appendix A Supplementary Materials

### Data and Code Availability

A subset of the code and processed dataset used in this study is provided in the Supplementary Materials. The remaining datasets are not publicly available at this stage due to ongoing collaborative studies but are available from the corresponding author upon reasonable request.
