# Supplementary material for: Physiology-informed LSTM framework integrating crop model and Sentinel-2 time series for rice nitrogen status estimation
Source: Plant Phenomics. 2026 Jun 5;8(2):100234. doi: 10.1016/j.plaphe.2026.100234 (PMC13293751; doi:10.1016/j.plaphe.2026.100234)
Supplement: Multimedia component 1 [file mmc1.docx]

# Appendix A. Supplementary materials

**A1. The study area**





Fig. S1. Study area and sampling locations. Panel (a) shows the geographic location of Xinghua City, Jiangsu Province, China, in the lower reaches of the Yangtze River. Panel (b) shows the spatial distribution of sampling sites in Xinghua City: yellow circles represent the 2,000 representative rice fields uniformly sampled across the cultivated area for DSSAT-based pseudo-label generation, while blue squares denote farm-scale field sampling sites where ground-based measurements were collected for model fine-tuning and validation.

**A2. Calibration and validation of the CERES-Rice model**

To ensure the reliability of DSSAT outputs used for pseudo-label generation, the CERES-Rice module was calibrated and validated using multi-year field observations. Model calibration employed experimental data from 2017 and 2018, while independent treatments from both years were reserved for validation. Calibration targeted cultivar-specific genetic coefficients to accurately reproduce rice phenology, biomass accumulation, nitrogen uptake, and grain yield under contrasting nitrogen levels and planting methods.

The calibration dataset included 2017 treatments under N rates of 0 and 270 kg/ha across three planting methods—direct seeding (DS), carpet seedling transplanting (TM), and pot seedling transplanting (BM)—and 2018 treatments under N rates of 135 and 405 kg/ha for both TM and BM transplanting. The remaining treatments were used for validation, including 2017 N rates of 135 and 405 kg/ha (DS, TM, BM) and 2018 N rates of 0 and 270 kg/ha (TM, BM). This cross-treatment, cross-year allocation provided a robust assessment of the model’s capacity to generalize beyond calibration conditions.

Parameter calibration followed the Generalized Likelihood Uncertainty Estimation (GLUE) framework (He et al., 2010), in which parameter sets were sampled using a Monte Carlo strategy (10,000 realizations per iteration). Phenological parameters were first calibrated to match observed development stages, after which yield-related genetic coefficients were optimized. For each sampled parameter set, likelihood values were computed based on the agreement between simulated and observed phenology, biomass, and yield, and posterior weights were used to identify behavioral parameter sets. The final calibrated coefficients were derived from the highest-likelihood ensemble.

Model performance was evaluated by comparing simulated and observed phenology and grain yield using the coefficient of determination (R^2^) and root mean square error (RMSE). For dynamic variables—leaf area index (LAI), plant dry matter (PDM), and plant nitrogen accumulation (PNA)—time-series trajectories were assessed using RMSE and the index of agreement (d), which quantifies the match between simulated and observed temporal patterns (Eq. (S1)). In addition, an overall R^2^ and RMSE were calculated using all calibration and validation samples combined to summarize model performance across the full range of nitrogen treatments and planting methods.

$$\begin{aligned} d=1-\frac{\sum_{i=1}^{n} \left( P_{i}-O_{i} \right)^{2}}{\sum_{i=1}^{n} \left( \left| P_{i}-\bar{O}_{i} \right|+\left| O_{i}-\bar{O}_{i} \right| \right)^{2}}\#\left( S1 \right) \end{aligned}$$

where $O_{i}$​ and $P_{i}$ are the observed and simulated target values, respectively; $\overline{O}$ is the mean of all observed values; and $n$ is the total number of observations. The index $d$ ranges from 0 to 1, with values closer to 1 indicating stronger agreement between model simulations and observations.

The validation results for phenology, grain yield, LAI, PDM, and PNA are presented in Figs. S2–S6, respectively.


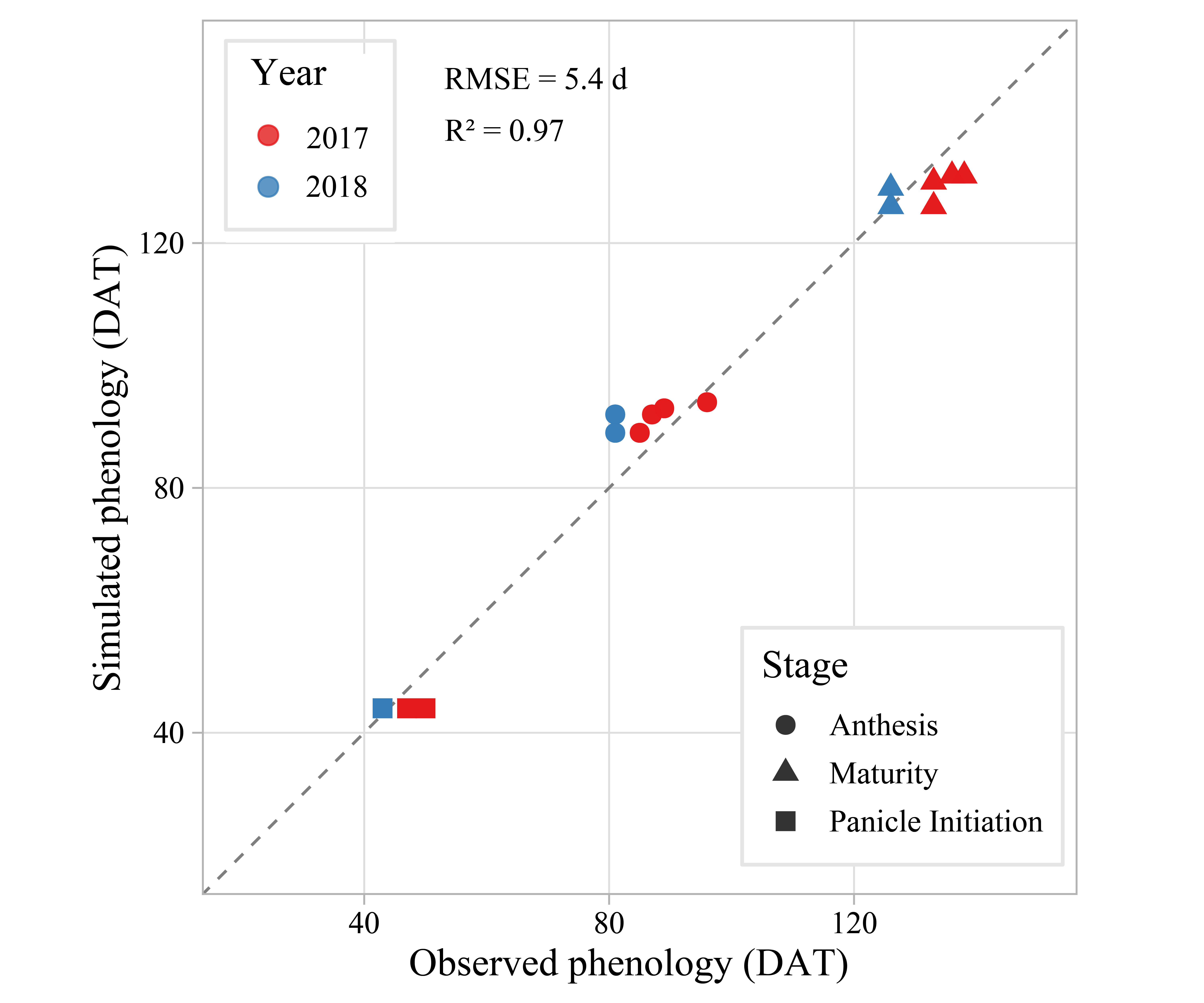


Fig. S2. Comparison between DSSAT-simulated and observed rice phenological stages during model validation. The dashed line represents the 1:1 relationship.


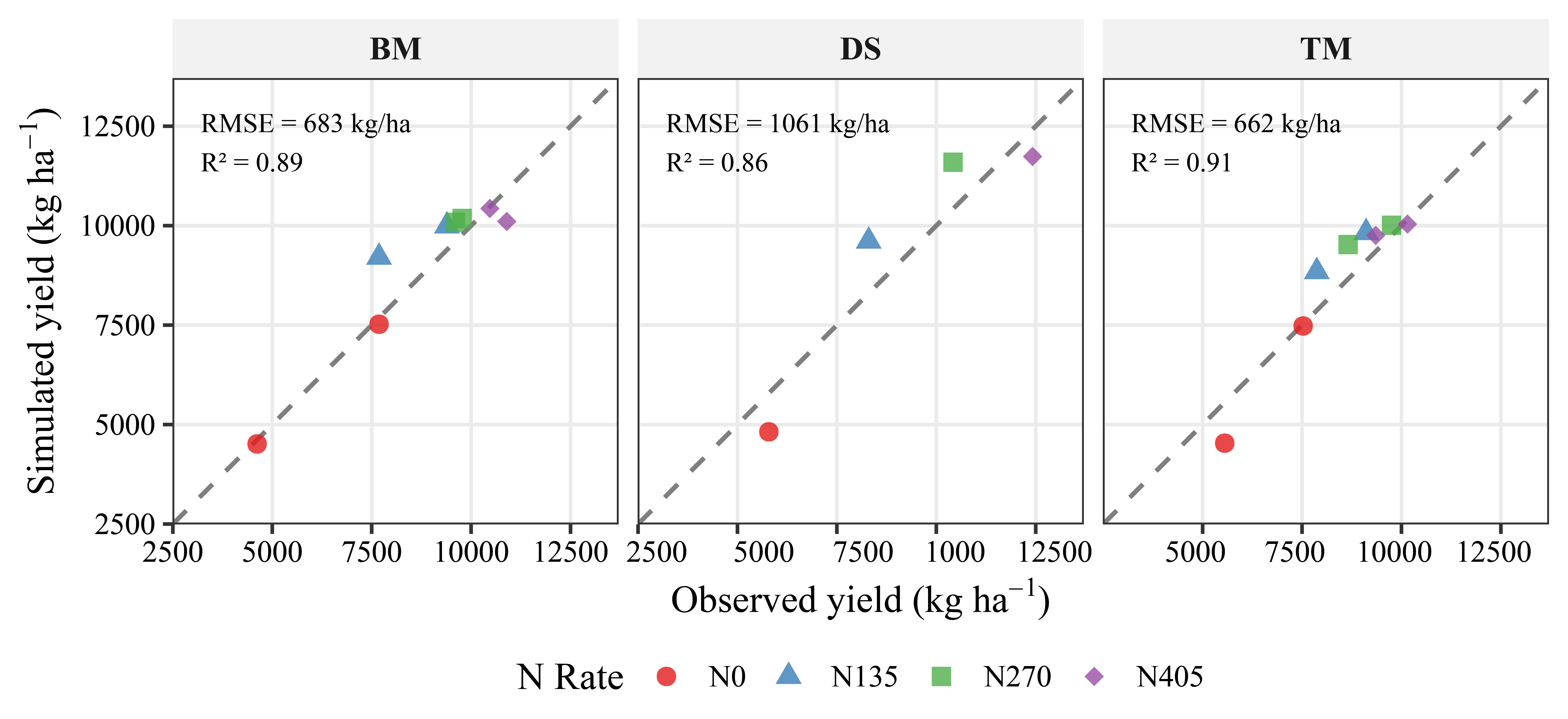
Fig. S3. Comparison between DSSAT-simulated and observed grain yield across nitrogen treatments and planting methods. TM, BM, and DS denote carpet seedling transplanting, pot seedling transplanting, and direct seeding, respectively; N0, N135, N270, and N405 indicate total nitrogen application rates of 0, 135, 270, and 405 kg/ha, respectively.





Fig. S4. Comparison of DSSAT-simulated and observed LAI dynamics. Blue lines represent simulated LAI trajectories, and red points indicate field observations. TM, BM, and DS denote carpet seedling transplanting, pot seedling transplanting, and direct seeding, respectively; N0, N135, N270, and N405 indicate total nitrogen application rates of 0, 135, 270, and 405 kg/ha, respectively.

 Fig. S5. Comparison of DSSAT-simulated PDM trajectories with observed PDM measurements. Blue lines indicate simulated values, and red points represent observations. TM, BM, and DS denote carpet seedling transplanting, pot seedling transplanting, and direct seeding, respectively; N0, N135, N270, and N405 indicate total nitrogen application rates of 0, 135, 270, and 405 kg/ha, respectively.



 Fig. S6. Comparison of DSSAT-simulated PNA trajectories with observed PNA measurements. Blue lines indicate simulated values, and red points represent observations. TM, BM, and DS denote carpet seedling transplanting, pot seedling transplanting, and direct seeding, respectively; N0, N135, N270, and N405 indicate total nitrogen application rates of 0, 135, 270, and 405 kg/ha, respectively.

**A3. Hyperparameter tuning and performance comparison of machine learning models**

To ensure a rigorous evaluation of the proposed framework against conventional approaches, we systematically optimized the hyperparameters of five standard machine learning algorithms: Random Forest (RF), XGBoost, Support Vector Machine (SVM), Neural Network (NNET), and Partial Least Squares regression (PLS). Hyperparameter tuning was performed using a grid search strategy coupled with 5-fold cross-validation. The selection criterion for the optimal parameter set was the minimization of the root mean square error (RMSE) for both PDM and PNA.

For models with high-dimensional hyperparameter spaces (XGBoost, SVM, and NNET), we examined the interaction between structural parameters (e.g., max_depth for XGBoost, hidden units for NNET) and regularization terms (e.g., learning rate, weight decay, or cost). For single-parameter models (RF and PLS), we analyzed the impact of model complexity, specifically the number of features per split (mtry) and the number of latent components (ncomp), on prediction error.

The hyperparameter tuning landscapes are visualized in Fig. S7. Heatmaps illustrate the sensitivity of PDM and PNA prediction accuracy to parameter interactions for multi-parameter models, while line charts demonstrate the error evolution for single-parameter models. The specific optimal hyperparameter configurations identified and used for the final model training are detailed in Table S1.

Table S1. Optimal hyperparameter configurations for PDM and PNA estimation across the five machine learning models.

| Model | Optimal Hyperparameters | |
| --- | --- | --- |
|  | PDM | PNA |
| RF | mtry = 2 | mtry = 2 |
| XGBoost | max_depth = 4  eta = 0.01 | max_depth = 4  eta = 0.01 |
| SVM | sigma = 0.1  C = 10 | sigma = 0.01  C = 100 |
| NNET | size = 15  decay = 0.001 | size = 10  decay = 0.1 |
| PLS | ncomp = 10 | ncomp = 10 |





Fig. S7. Hyperparameter tuning results for the five machine learning baselines (RF, PLS, XGBoost, SVM, and NNET) applied to PDM and PNA estimation. Line plots (top row) display the RMSE variation against a single hyperparameter for RF and PLS. Heatmaps (middle and bottom rows) illustrate the interaction effects of dual hyperparameters on RMSE for XGBoost, SVM, and NNET. The points (or circles) within the plots indicate the optimal parameter combinations that minimized the RMSE.

**A4. PI-LSTM configuration, model comparison, and statistical testing**

The main architectural, pretraining, and fine-tuning hyperparameters of the PI-LSTM model are summarized in Table S2. Model comparison results are presented in Table S3, and statistical significance tests based on the five-fold cross-validation results are provided in Table S4.

Table S2. Summary of PI-LSTM architecture, pretraining settings, and fine-tuning hyperparameter selection.

| Component | Hyperparameter / setting | Value / selection strategy |
| --- | --- | --- |
| Model architecture | LSTM layers | Two stacked LSTM layers |
|  | Hidden units | 128 hidden units per layer |
| Pretraining | Batch size | 64 |
|  | Pretraining epochs | 50 |
| Fine-tuning | Trainable layers | Upper recurrent layer and final fully connected layer |
|  | Frozen layers | Lower recurrent layers retained from pretraining |
|  | Learning rate | Selected by field-level five-fold cross-validation; final value = 0.0001 |
|  | Fine-tuning epochs | Selected by field-level five-fold cross-validation; final value = 30 |
| Model selection | Selection criterion | Lowest mean validation RMSE averaged across folds |

Beyond the conventional machine learning baselines, the key hyperparameters of the PI-LSTM fine-tuning stage were selected under the same field-level five-fold cross-validation framework used in the main analysis. The tuning focused on the learning rate and number of fine-tuning epochs, and model selection was based on the minimum validation RMSE averaged across folds. Based on this procedure, the final fine-tuning settings were set to a learning rate of 0.0001 and 30 epochs. Other architectural and pretraining settings, including two stacked LSTM layers, 128 hidden units per layer, a batch size of 64, and 50 pretraining epochs, were fixed before field fine-tuning and are summarized in Table S2. The resulting performance comparison between the PI-LSTM and baseline models is presented in Table S3.

Table S3. Performance comparison of different machine learning models for PDM and PNA prediction.

| Target variable | Model algorithm | RMSE | R^2^ | RRMSE (%) |
| --- | --- | --- | --- | --- |
| PDM | LSTM | 955.43 | 0.87 | 15.30 |
|  | SVM | 1,017.31 | 0.86 | 16.42 |
|  | RF | 1,050.48 | 0.85 | 16.95 |
|  | PLS | 1,073.13 | 0.85 | 17.32 |
|  | XGB | 1,074.07 | 0.85 | 17.33 |
|  | NNET | 1,123.15 | 0.83 | 18.13 |
| PNA | LSTM | 12.79 | 0.83 | 10.30 |
|  | SVM | 15.69 | 0.81 | 12.54 |
|  | RF | 16.03 | 0.81 | 12.82 |
|  | NNET | 16.15 | 0.81 | 12.91 |
|  | PLS | 16.22 | 0.80 | 12.97 |
|  | XGB | 16.46 | 0.80 | 13.15 |

To further evaluate whether the observed performance differences were statistically meaningful, statistical significance analyses were conducted based on the five-fold cross-validation results for both PDM and PNA. Specifically, Friedman tests were first used to assess the overall differences among the six models for each metric, followed by post-hoc paired Wilcoxon signed-rank tests comparing the proposed LSTM model with each baseline model. Holm correction was applied to adjust for multiple comparisons.

As shown in Table S4, significant overall differences among models were detected for all four metrics in both datasets. For PDM, the Friedman tests were significant for RMSE (p = 0.01166), MAE (p = 0.01341), R² (p = 0.006002), and RRMSE (p = 0.0128). For PNA, the corresponding tests were also significant for RMSE (p = 0.005454), MAE (p = 0.001329), R² (p = 0.02037), and RRMSE (p = 0.006296). These results indicate that model choice had a statistically significant overall effect on predictive performance in both tasks.

Post-hoc paired comparisons further showed that LSTM consistently achieved lower average RMSE, MAE, and RRMSE, and higher average R² than most baseline models, especially for PNA. However, after Holm correction, not all pairwise differences remained statistically significant. This may be partly attributable to the limited statistical power associated with the five-fold design and should therefore be interpreted with caution. Overall, the significance analysis supports the conclusion that LSTM provided the strongest average predictive performance, while also indicating that caution is needed when interpreting pairwise significance at the fold level.

Table S4. Statistical significance analysis of model comparisons for PDM and PNA based on five-fold cross-validation.

A. Overall model differences (Friedman tests)

| Target | Metric | Chi-squared | df | p-value |
| --- | --- | --- | --- | --- |
| PDM | RMSE | 14.714 | 5 | 0.0117 |
|  | R² | 16.314 | 5 | 0.0060 |
|  | RRMSE | 14.486 | 5 | 0.0128 |
| PNA | RMSE | 16.543 | 5 | 0.0055 |
|  | R² | 13.343 | 5 | 0.0204 |
|  | RRMSE | 16.200 | 5 | 0.0063 |

B. Post-hoc paired Wilcoxon signed-rank tests comparing LSTM with each baseline model

| Target | Metric | Baseline | LSTM mean | Baseline mean | Mean difference | Holm-adjusted p |
| --- | --- | --- | --- | --- | --- | --- |
| PDM | RMSE | PLS | 945.2847 | 1077.7273 | -132.4426 | 1.00000 |
|  |  | NNET | 945.2847 | 1227.2787 | -281.9940 | 0.15625 |
|  |  | RF | 945.2847 | 981.2766 | -35.9919 | 1.00000 |
|  |  | SVM | 945.2847 | 976.9767 | -31.6920 | 1.00000 |
|  |  | XGB | 945.2847 | 964.4007 | -19.1160 | 1.00000 |
|  |  | NNET | 686.8929 | 913.6673 | -226.7744 | 0.31250 |
|  |  | RF | 686.8929 | 659.4925 | 27.4005 | 1.00000 |
|  |  | SVM | 686.8929 | 642.7023 | 44.1906 | 1.00000 |
|  |  | XGB | 686.8929 | 648.6820 | 38.2109 | 1.00000 |
|  | R^2^ | PLS | 0.8736 | 0.8385 | 0.0351 | 1.00000 |
|  |  | NNET | 0.8736 | 0.7840 | 0.0896 | 0.15625 |
|  |  | RF | 0.8736 | 0.8706 | 0.0030 | 1.00000 |
|  |  | SVM | 0.8736 | 0.8742 | -0.0006 | 1.00000 |
|  |  | XGB | 0.8736 | 0.8737 | -0.0001 | 1.00000 |
|  | RRMSE | PLS | 0.1512 | 0.1731 | -0.0218 | 0.62500 |
|  |  | NNET | 0.1512 | 0.1972 | -0.0460 | 0.15625 |
|  |  | RF | 0.1512 | 0.1577 | -0.0065 | 1.00000 |
|  |  | SVM | 0.1512 | 0.1571 | -0.0058 | 1.00000 |
|  |  | XGB | 0.1512 | 0.1550 | -0.0038 | 1.00000 |
| PNA | RMSE | PLS | 12.3589 | 16.1997 | -3.8408 | 0.15625 |
|  |  | NNET | 12.3589 | 15.5236 | -3.1648 | 0.15625 |
|  |  | RF | 12.3589 | 14.8628 | -2.5039 | 0.15625 |
|  |  | SVM | 12.3589 | 14.5679 | -2.2090 | 0.15625 |
|  |  | XGB | 12.3589 | 14.4333 | -2.0744 | 0.15625 |
|  |  | NNET | 9.5262 | 12.0854 | -2.5592 | 0.15625 |
|  |  | RF | 9.5262 | 11.6511 | -2.1249 | 0.15625 |
|  |  | SVM | 9.5262 | 11.2399 | -1.7137 | 0.15625 |
|  |  | XGB | 9.5262 | 11.2300 | -1.7038 | 0.15625 |
|  | R^2^ | PLS | 0.8669 | 0.7884 | 0.0785 | 0.31250 |
|  |  | NNET | 0.8669 | 0.8121 | 0.0548 | 0.31250 |
|  |  | RF | 0.8669 | 0.8264 | 0.0404 | 0.31250 |
|  |  | SVM | 0.8669 | 0.8364 | 0.0305 | 0.31250 |
|  |  | XGB | 0.8669 | 0.8374 | 0.0295 | 0.31250 |
|  | RRMSE | PLS | 0.0996 | 0.1284 | -0.0288 | 0.15625 |
|  |  | NNET | 0.0996 | 0.1240 | -0.0244 | 0.15625 |
|  |  | RF | 0.0996 | 0.1187 | -0.0191 | 0.15625 |
|  |  | SVM | 0.0996 | 0.1164 | -0.0167 | 0.15625 |
|  |  | XGB | 0.0996 | 0.1153 | -0.0157 | 0.15625 |

Note: Friedman tests were used to assess the overall differences among the six models for each metric. Post-hoc one-sided paired Wilcoxon signed-rank tests were then performed to compare the proposed LSTM model with each baseline model. Holm-adjusted p-values were used to account for multiple comparisons. For RMSE and RRMSE, smaller values indicate better performance, whereas for R², larger values indicate better performance. Mean difference was calculated as LSTM minus baseline; therefore, negative values indicate better LSTM performance for RMSE, MAE, and RRMSE, while positive values indicate better LSTM performance for R^2^.

**A5. Temporal consistency example and regional applicability of the fine-tuned LSTM model**

To further evaluate the mechanistic fidelity and spatial generalization capability of the proposed framework, we examined both representative temporal growth trajectories and regional-scale spatial patterns. Fig. S8 illustrates the agreement between DSSAT-simulated and LSTM-predicted daily PDM and PNA trajectories, demonstrating that the fine-tuned model preserves physiologically meaningful growth dynamics. Fig. S9 presents regional-scale maps of rice growth status at the jointing stage across the Xinghua region, highlighting the model’s ability to upscale field-level predictions to spatially continuous monitoring of biomass and nitrogen accumulation.


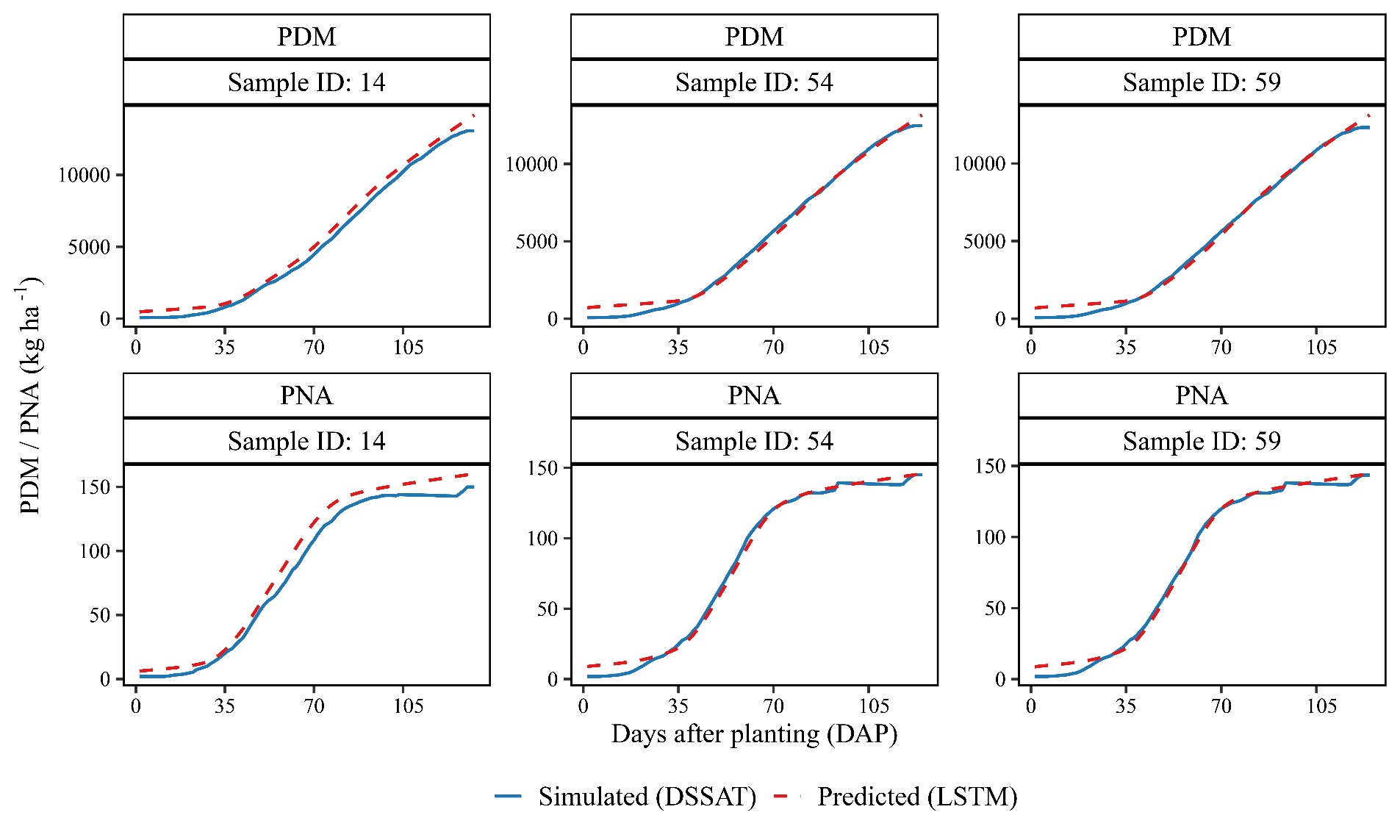


Fig. S8. Representative growth trajectories demonstrating mechanistic consistency between DSSAT and LSTM predictions.


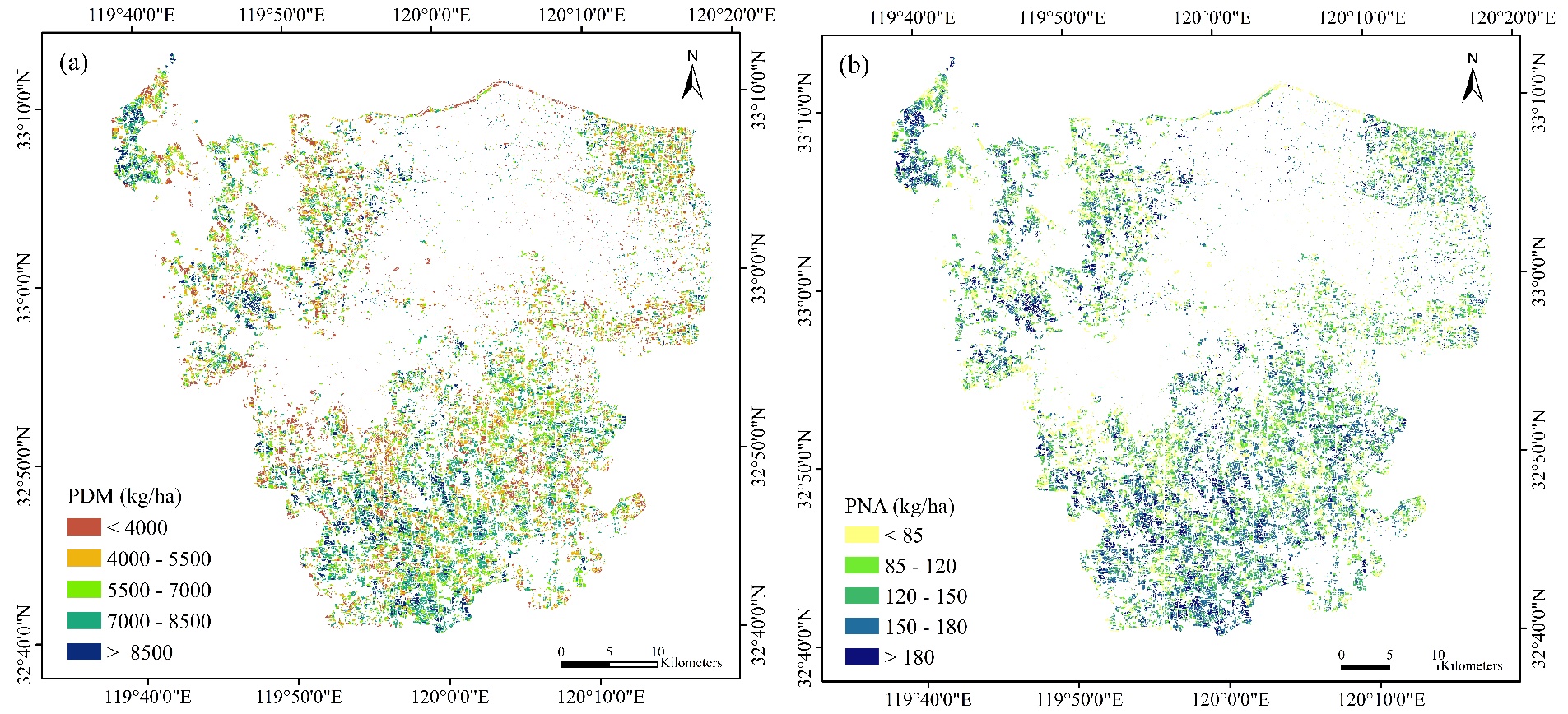


Fig. S9. Regional-scale maps of rice growth status for (a) PDM and (b) PNA at the jointing stage across the Xinghua region, estimated by the fine-tuned LSTM.

**A6. Sensitivity analysis of matching weights**

To evaluate whether the pseudo-label reconstruction was sensitive to the weighting factors in the composite matching score, we conducted a sensitivity analysis around the baseline setting ((wr, wc, wd) = (0.4, 0.4, 0.2)). Correlation and cosine similarity were assigned relatively larger and equal weights because they represent trend agreement and curve-shape similarity between observed and simulated trajectories, whereas the DTW-based term was treated as a complementary indicator of temporal alignment after normalization.


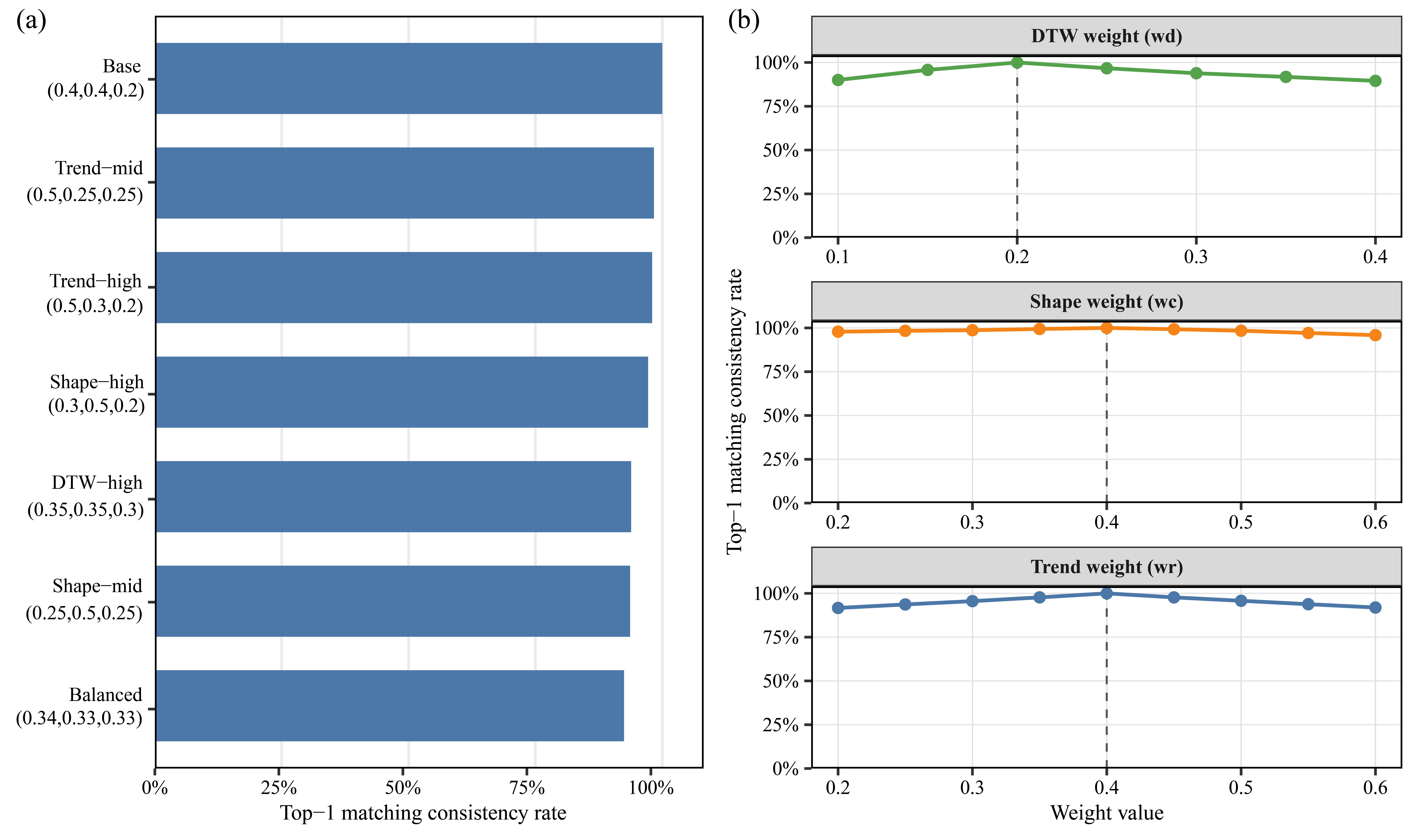


Fig. S10. Sensitivity analysis of the matching results under alternative weighting schemes. (a) Consistency rate of top-1 matched scenarios under representative weighting schemes relative to the baseline setting. (b) One-factor-at-a-time sensitivity curves showing how top-1 matching consistency changes when each weight is varied, with the remaining weights proportionally adjusted to satisfy (wr + wc + wd = 1). Dashed vertical lines indicate the baseline values.

One-factor-at-a-time perturbations were performed while maintaining the constraint (wr + wc + wd = 1). The consistency rate of the top-ranked matched scenario relative to the baseline remained high across all tested combinations: 91.7%–97.7% when varying (wr), 95.9%–99.4% when varying (wc), and 89.5%–96.7% when varying (wd). Across several representative weighting schemes, the consistency rate ranged from 92.5% to 98.4%.

These results suggest that the matching outcomes were generally robust to moderate changes in the weighting factors, supporting the use of ((0.4, 0.4, 0.2)) as a reasonable baseline setting rather than a unique optimum.

**A7. Algorithmic workflow and reproducibility code**

To improve reproducibility, the main computational workflow of the proposed framework is summarized below. The algorithmic descriptions cover DSSAT-based pseudo-label construction, daily sequence generation, PI-LSTM pretraining, transfer learning, and evaluation. The accompanying code blocks focus on the PI-LSTM implementation, including pretraining, field-level five-fold fine-tuning, cross-validation, and performance evaluation. The earlier dataset-construction steps, including CERES-Rice calibration and validation, DSSAT scenario library generation, satellite–DSSAT trajectory matching, and pseudo-label dataset construction, are summarized algorithmically rather than provided as complete executable scripts.

**Configuration, variables, and preprocessing**

# Code Block S1. Configuration, variables, and data preprocessing

import os

import copy

import numpy as np

import pandas as pd

import torch

import torch.nn as nn

from torch.utils.data import Dataset, DataLoader

from torch.nn.utils.rnn import pad_sequence, pack_padded_sequence, pad_packed_sequence

from sklearn.preprocessing import StandardScaler, MinMaxScaler

from sklearn.model_selection import KFold

from sklearn.metrics import r2_score, mean_squared_error

# -----------------------------

# Configuration

# -----------------------------

PRETRAIN_CSV = "LSTM_DSSAT_Pretrain_Data.csv"

FIELD_DATA = "dataset_for_fine_tuning.xlsx"

BATCH_SIZE = 64

HIDDEN_DIM = 128

NUM_LAYERS = 2

PRETRAIN_LR = 0.001

PRETRAIN_EPOCHS = 50

FT_LR = 0.0001

FT_EPOCHS = 30

DEVICE = torch.device("cuda" if torch.cuda.is_available() else "cpu")

# -----------------------------

# Input and target variables

# -----------------------------

SCALE_FEATURES = [

"NDVI_filled", "NDRE_filled", "GNDVI_filled", "CIRE_filled", "RESAVI_filled",

"TAVD", "TMXD", "TMND", "SRAD", "PRED",

"cum_gdd", "cum_rain", "cum_srad", "Nbase", "DAP"

]

INPUT_COLS = SCALE_FEATURES

TARGET_COLS = ["PDM", "PNA"]

MASK_COLS = ["mask_target_pdm", "mask_target_pna"]

# -----------------------------

# Read and clean DSSAT pseudo-label data

# -----------------------------

df = pd.read_csv(PRETRAIN_CSV)

df = df.replace([np.inf, -np.inf], np.nan)

df[INPUT_COLS] = df[INPUT_COLS].fillna(0)

df[TARGET_COLS] = df[TARGET_COLS].fillna(0)

df[MASK_COLS] = df[MASK_COLS].fillna(0)

# Example split for model development. For the final manuscript results,

# use leave-one-year-out validation across 2020-2024 pseudo-label data.

train_df = df[df["Year"] < 2024].copy()

test_df = df[df["Year"] >= 2024].copy()

# Fit scalers only on the training subset to avoid information leakage.

x_scaler = StandardScaler()

x_scaler.fit(train_df[SCALE_FEATURES])

train_df[SCALE_FEATURES] = x_scaler.transform(train_df[SCALE_FEATURES])

test_df[SCALE_FEATURES] = x_scaler.transform(test_df[SCALE_FEATURES])

y_scaler = MinMaxScaler(feature_range=(0, 1))

y_scaler.fit(train_df[TARGET_COLS])

train_df[TARGET_COLS] = y_scaler.transform(train_df[TARGET_COLS])

test_df[TARGET_COLS] = y_scaler.transform(test_df[TARGET_COLS])

**Sequence dataset and padding**

# Code Block S2. Daily sequence dataset and padding function

class RiceDataset(Dataset):

"""Create one daily sequence for each field-year."""

def __init__(self, data_frame):

self.sequences = []

data_frame = data_frame.sort_values(["field_id", "Year", "DATE"])

grouped = data_frame.groupby(["field_id", "Year"])

for _, group in grouped:

x = group[INPUT_COLS].values.astype(np.float32)

y = group[TARGET_COLS].values.astype(np.float32)

mask = group[MASK_COLS].values.astype(np.float32)

x = np.nan_to_num(x, nan=0.0)

y = np.nan_to_num(y, nan=0.0)

mask = np.nan_to_num(mask, nan=0.0)

self.sequences.append((x, y, mask))

def __len__(self):

return len(self.sequences)

def __getitem__(self, idx):

return self.sequences[idx]

def collate_fn(batch):

"""Pad variable-length field-year sequences within each mini-batch."""

batch.sort(key=lambda x: len(x[0]), reverse=True)

xs, ys, masks = zip(*batch)

lengths = torch.tensor([len(x) for x in xs])

xs = [torch.from_numpy(x) for x in xs]

ys = [torch.from_numpy(y) for y in ys]

masks = [torch.from_numpy(m) for m in masks]

return (

pad_sequence(xs, batch_first=True),

pad_sequence(ys, batch_first=True),

pad_sequence(masks, batch_first=True),

lengths,

)

train_loader = DataLoader(RiceDataset(train_df), batch_size=BATCH_SIZE,

shuffle=True, collate_fn=collate_fn)

test_loader = DataLoader(RiceDataset(test_df), batch_size=BATCH_SIZE,

shuffle=False, collate_fn=collate_fn)

**PI-LSTM and mask-aware loss**

# Code Block S3. PI-LSTM model and mask-aware loss

class PILSTM(nn.Module):

"""Two-layer LSTM with a fully connected output layer for PDM and PNA."""

def __init__(self, input_dim, hidden_dim, num_layers, output_dim):

super().__init__()

self.lstm = nn.LSTM(

input_size=input_dim,

hidden_size=hidden_dim,

num_layers=num_layers,

batch_first=True,

)

self.fc = nn.Linear(hidden_dim, output_dim)

def forward(self, x, lengths):

packed_x = pack_padded_sequence(

x, lengths.cpu(), batch_first=True, enforce_sorted=False

)

packed_out, _ = self.lstm(packed_x)

out, _ = pad_packed_sequence(packed_out, batch_first=True)

pred = self.fc(out)

return pred

model = PILSTM(

input_dim=len(INPUT_COLS),

hidden_dim=HIDDEN_DIM,

num_layers=NUM_LAYERS,

output_dim=len(TARGET_COLS),

).to(DEVICE)

criterion = nn.MSELoss(reduction="none")

def masked_mse_loss(pred, target, mask):

"""Compute MSE only at valid target time steps."""

loss = criterion(pred, target)

loss = loss * mask

return loss.sum() / (mask.sum() + 1e-8)

**DSSAT-based pretraining**

# Code Block S4. DSSAT-based pretraining

optimizer = torch.optim.Adam(model.parameters(), lr=PRETRAIN_LR)

for epoch in range(PRETRAIN_EPOCHS):

model.train()

epoch_loss = 0.0

steps = 0

for x, y, mask, lengths in train_loader:

x = x.to(DEVICE)

y = y.to(DEVICE)

mask = mask.to(DEVICE)

optimizer.zero_grad()

pred = model(x, lengths)

loss = masked_mse_loss(pred, y, mask)

loss.backward()

optimizer.step()

epoch_loss += loss.item()

steps += 1

if (epoch + 1) % 5 == 0:

print(f"Epoch {epoch+1:03d}/{PRETRAIN_EPOCHS}, loss={epoch_loss/max(steps,1):.6f}")

# Save pretrained weights for transfer learning.

pretrained_state = copy.deepcopy(model.state_dict())

**Field-level five-fold transfer learning**

# Code Block S5. Field-level five-fold transfer learning

# Read observed field data from Exp. 2 and apply the scalers fitted during pretraining.

df_real = pd.read_excel(FIELD_DATA)

if "date" in df_real.columns:

df_real = df_real.rename(columns={"date": "DATE"})

df_real = df_real.replace([np.inf, -np.inf], np.nan)

df_real[INPUT_COLS] = df_real[INPUT_COLS].fillna(0)

df_real[TARGET_COLS] = df_real[TARGET_COLS].fillna(0)

df_real[MASK_COLS] = df_real[MASK_COLS].fillna(0)

df_real[SCALE_FEATURES] = x_scaler.transform(df_real[SCALE_FEATURES])

df_real[TARGET_COLS] = y_scaler.transform(df_real[TARGET_COLS])

all_field_ids = df_real["field_id"].unique()

kf = KFold(n_splits=5, shuffle=True, random_state=42)

cv_preds, cv_trues, cv_masks = [], [], []

for fold, (train_idx, val_idx) in enumerate(kf.split(all_field_ids), start=1):

train_ids = all_field_ids[train_idx]

val_ids = all_field_ids[val_idx]

df_train_fold = df_real[df_real["field_id"].isin(train_ids)].copy()

df_val_fold = df_real[df_real["field_id"].isin(val_ids)].copy()

dl_train = DataLoader(RiceDataset(df_train_fold), batch_size=8,

shuffle=True, collate_fn=collate_fn)

dl_val = DataLoader(RiceDataset(df_val_fold), batch_size=8,

shuffle=False, collate_fn=collate_fn)

curr_model = PILSTM(len(INPUT_COLS), HIDDEN_DIM, NUM_LAYERS,

len(TARGET_COLS)).to(DEVICE)

curr_model.load_state_dict(pretrained_state)

# Optional manuscript-aligned freezing strategy:

# Freeze the lower recurrent layer and fine-tune the upper recurrent layer and FC layer.

for name, param in curr_model.lstm.named_parameters():

if name.startswith("weight_ih_l0") or name.startswith("weight_hh_l0") or \

name.startswith("bias_ih_l0") or name.startswith("bias_hh_l0"):

param.requires_grad = False

optimizer = torch.optim.Adam(

filter(lambda p: p.requires_grad, curr_model.parameters()), lr=FT_LR

)

for epoch in range(FT_EPOCHS):

curr_model.train()

for x, y, mask, lengths in dl_train:

x = x.to(DEVICE)

y = y.to(DEVICE)

mask = mask.to(DEVICE)

optimizer.zero_grad()

pred = curr_model(x, lengths)

loss = masked_mse_loss(pred, y, mask)

loss.backward()

optimizer.step()

curr_model.eval()

with torch.no_grad():

for x, y, mask, lengths in dl_val:

x = x.to(DEVICE)

pred = curr_model(x, lengths)

p_np = pred.cpu().numpy()

y_np = y.numpy()

m_np = mask.numpy()

lengths_np = lengths.numpy()

for i in range(x.shape[0]):

L = lengths_np[i]

cv_preds.append(p_np[i, :L, :])

cv_trues.append(y_np[i, :L, :])

cv_masks.append(m_np[i, :L, :])

**Evaluation and export**

# Code Block S6. Evaluation and export of predictions

flat_p = np.concatenate(cv_preds, axis=0)

flat_t = np.concatenate(cv_trues, axis=0)

flat_m = np.concatenate(cv_masks, axis=0)

# Convert normalized values back to original units.

real_p = y_scaler.inverse_transform(flat_p)

real_t = y_scaler.inverse_transform(flat_t)

def evaluate_variable(variable_index, variable_name):

valid_idx = flat_m[:, variable_index] > 0.5

observed = real_t[valid_idx, variable_index]

predicted = real_p[valid_idx, variable_index]

r2 = r2_score(observed, predicted)

rmse = np.sqrt(mean_squared_error(observed, predicted))

nrmse = rmse / np.mean(observed) * 100

return {

"Variable": variable_name,

"R2": r2,

"RMSE": rmse,

"nRMSE_percent": nrmse,

}

metrics = [

evaluate_variable(0, "PDM"),

evaluate_variable(1, "PNA"),

]

metrics_df = pd.DataFrame(metrics)

metrics_df.to_csv("field_level_cv_metrics.csv", index=False)

# Export observed and predicted values at valid target time steps.

export_list = []

for i, variable_name in enumerate(["PDM", "PNA"]):

valid_idx = flat_m[:, i] > 0.5

export_list.append(pd.DataFrame({

"Variable": variable_name,

"Observed": real_t[valid_idx, i],

"Predicted": real_p[valid_idx, i],

}))

pd.concat(export_list, ignore_index=True).to_csv(

"field_level_cv_predictions.csv", index=False

)

**Reference**

He, J., Jones, J.W., Graham, W.D., Dukes, M.D., 2010. Influence of likelihood function choice for estimating crop model parameters using the generalized likelihood uncertainty estimation method. Agricultural Systems. 103(5), 256-264. <https://doi.org/https://doi.org/10.1016/j.agsy.2010.01.006>.
